# Psychosocial correlates of physical activity in cancer survivors: a systematic review and meta-analysis

**DOI:** 10.1007/s11764-024-01559-6

**Published:** 2024-03-06

**Authors:** Bruno Rodrigues, Jorge Encantado, Sofia Franco, Marlene N. Silva, Eliana V. Carraça

**Affiliations:** 1 Faculty of Sport, University of Porto (Research Centre in Physical Activity, Health and Leisure), R. Dr. Plácido da Costa 91, 4200-450 Porto, Portugal; 2 Faculdade de Motricidade Humana, CIPER, Universidade de Lisboa, Cruz Quebrada, Portugal; 3 CIDEFES, Universidade Lusófona, Lisboa & CIFI2D, Universidade do Porto, Porto, Universidade Lusófona and Universidade do Porto, Lisbon and Porto, Portugal

**Keywords:** Physical activity, Adherence, Psychosocial correlates, Cancer survivors

## Abstract

**Background:**

Physical activity (PA) is a non-pharmacological approach to optimize health benefits in cancer survivors and is recommended as part of care. However, most cancer survivors fail to meet PA recommendations. The current systematic review and meta-analysis aimed to identify psychosocial correlates of free-living PA in cancer survivors.

**Methods:**

Three electronic databases were searched (PubMed, PsycINFO, and SportDiscus). Meta-analyses were conducted for psychosocial correlates tested ≥ 3 times.

**Results:**

Sixty-four articles were included. Eighty-eight different free-living PA correlates were identified. Meta-analyses (*n* = 32 studies) tested 23 PA correlates, of which 16 were significant (*p* < 0.05). Larger effect sizes (0.30 < ES > 0.45) were found for exercise self-efficacy, perceived behavioral control, intention, lower perceived barriers for exercise, enjoyment, perceived PA benefits, and attitudes. Small-to-moderate effects (0.18 < ES < 0.22) were found for subjective norms, physical functioning, quality of life, depression, and mental health. These findings were generally in line with narrative results.

**Conclusions:**

This systematic review highlights important psychosocial correlates of free-living PA that can be targeted in future PA promotion interventions for cancer survivors. Constructs mainly from SCT and TPB were the most studied and appear to be associated with free-living PA in this population. However, we cannot currently assert which frameworks might be more effective. Further studies of better methodological quality, per correlate and theory, exploring longer-term associations and across different types of cancer, are needed.

**Implications for Cancer Survivors:**

Having higher exercise self-efficacy, perceived behavioral control, intention, enjoyment and perceived PA benefits, more positive attitudes towards PA, and lower perceived barriers for exercise, can help increase PA in cancer survivors.

**Supplementary Information:**

The online version contains supplementary material available at 10.1007/s11764-024-01559-6.

## Introduction

Cancer is expected to become the leading cause of death in the near future [1]. Concurrently, there was an increase in survivorship rates due to advances in cancer detection and treatment, which brought new challenges to cancer care [1]. Despite advances in treatment, cancer survivors (i.e., from diagnosis to the end of life) still face several treatment-related late effects (physical and psychological problems), increased risk of cancer recurrence, and higher vulnerability to chronic diseases [2].

Physical activity (PA; i.e., any bodily movement that results in energy expenditure [3]) is being progressively considered as a nonpharmacologic way to optimize health benefits and outcomes in cancer survivors [4–8], and is recommended as part of care [9–12]. The evidence is clear regarding the contribution to improved cardiorespiratory capacity, muscle strength, quality of life, sleep, fatigue, and depression [6, 8, 13–16]. Also, PA seems to reduce the risk of recurrence [4] and cardiovascular disease [17], reduce mortality from cancer and from any cause [8], and improve the effectiveness and tolerance to anticancer treatment [18]. Guidelines for aerobic PA advocate that cancer survivors should do at least 150 min/week of moderate-intensity PA, or 75 min/week of vigorous-intensity PA, or an equivalent combination of both intensities [12]. Strength exercises should be performed at least 2 days a week, involving the main muscle groups [12]. Free-living PA refers to physical activities performed in the context of daily, family, and community activities for purposes of leisure (walking, gardening, swimming, sport, and dance), commuting (cycling or walking), occupation/labor, or planned exercise [19–21]. Adherence to free-living PA can be defined as a dose of PA consistent with the abovementioned PA recommendations. However, most cancer survivors fail to adhere to these recommendations [22–24], due to barriers such as lack of motivation, time, and interest, or to symptoms associated with the disease and/or treatment, with the most pervasive being fatigue and pain [25, 26].

### Psychosocial correlates of free-living PA

Understanding the psychosocial correlates of increased free-living PA is crucial to increase compliance with PA recommendations, and ultimately, cope with cancer-related symptoms and side effects from treatment. A previous review has found that exercise intention, exercise stage of change, and perceived behavioral control were significantly associated with increased adherence to exercise programs in cancer survivors [27], while another review reported inconsistent findings for these same psychological factors, as well as for self-efficacy, extraversion, attitude, fatigue, and quality of life [23]. Inconsistent results were also found for psychological correlates of exercise maintenance (in free-living conditions), namely self-efficacy, attitudes (instrumental and affective), fatigue, quality of life, and intention [23]. Hence, there is still insufficient knowledge about psychosocial factors that may facilitate or hinder exercise/PA participation in free-living conditions in cancer survivors (especially in the long-term/exercise maintenance). A deeper insight into this matter may contribute to an earlier identification of survivors that are more resistant to the adoption of PA, to a better matching between survivors’ psychosocial characteristics and available interventions, to a better allocation of resources, and to the design of more effective interventions. Furthermore, exercise/PA correlates may be different across different phases of survivorship [24], but knowledge about which factors are more relevant in different phases of survivorship, as well as in different cancer types, remains scarce.

### Main theoretical frameworks of PA correlates

Theoretical frameworks are valuable assets when predicting health behavior change and adherence, as they provide guidance on what needs to be changed (i.e., over which correlates to intervene), how it can be changed (i.e., using which behavior change techniques), and why the intervention has worked (i.e., through which pathways). Still, early research in the field of PA adherence was mainly concerned with the identification of associations between a very diverse (atheoretical) set of variables and PA adherence, disregarding its interrelations at a theoretical level. Multiple theories have since been used to better understand the complexity behind PA adherence, and how to successfully foster it [28]. The most common frameworks pertain to the Theory of Planned Behavior (TPB), Social Cognitive Theory (SCT), the Transtheoretical Model (TTM), and Self-Determination Theory (SDT). TPB [29], widely used in several interventions [30–33], poses that one’s intention to perform the behavior (and subsequently, one’s likelihood of engaging in it) is driven by the degree to which a behavior is positively or negatively valued (affective attitudes), the evaluation of the behavior’s consequences (instrumental attitudes), one’s beliefs of which behaviors are typically performed by significant others (social descriptive norms), one’s beliefs of which behaviors are typically approved or disapproved in society (injunctive norms), and by the perceived ease or difficulty of performing such behavior (perceived behavioral control). SCT [34] has also been applied in several interventions [32, 33, 35–37], and is largely based on the enhancement of self-efficacy (i.e., one’s belief on his/her own ability to perform the behavior), which is positively associated with other SCT elements, namely the beliefs about what the behavior will bring about (outcome expectations), the person’s behavior and outcome goals, and perceived barriers (i.e., perceived impediments to perform the behavior). Social support, i.e., the physical and emotional comfort provided by others, is also an important element in SCT, increasing the likelihood of behavior adoption. TTM [38], applied in different interventions [32, 33, 39], is an integrative model proposing that people move through different stages of motivational readiness to adhere to a behavior (precontemplation, contemplation, preparation, action, and maintenance), using multiple cognitive (e.g., decisional balance) and behavioral (e.g., action plans, self-monitoring) strategies. SDT [40, 41] has also been showing promising results mainly in the prediction of sustained (long-term) adherence to PA and other health behaviors [42, 43], although it has been somewhat less explored in cancer populations [44, 45]. According to SDT, need-supportive environments are required to satisfy one’s basic psychological needs for autonomy (sense of personal choice and volition), competence (perception of effectively interacting with the environment) and relatedness (sense of belonging and respect), and in turn foster autonomous (better quality) motivations, as opposed to controlled motivations (based on internal and external pressures) or amotivation. Autonomous motivations have been shown to result in more favorable psychological outcomes and lasting adherence to PA [42, 46, 47]. Given the diversity of frameworks involved, knowing which theoretical constructs (from specific theoretical frameworks) might be more useful to change cancer survivors PA behaviors is still undetermined.

The current systematic review and meta-analysis aimed to identify and summarize psychosocial correlates of free-living PA in cancer survivors, which are typically measured with self-reported (subjective) psychosocial questionnaires, and likely to influence an individual psychologically and/or socially.

## Methods

This systematic review and meta-analysis followed the PRISMA statement for reporting systematic reviews [48].

### Eligibility criteria

To be included, studies had to comply with the following inclusion criteria: include adults (above 18 years); cancer survivors (i.e., from the time of diagnosis to the end of life) [49]; experimental and observational research designs that evaluate associations between at least one psychosocial correlate and PA levels (either objectively or subjectively measured), or test differences in psychosocial variables between active and inactive groups of cancer survivors. Interventions involving exercise (aerobic, strength, combined, multicomponent) or lifestyle interventions promoting changes in free-living PA were included. Putative correlates were self-reported (questionnaire-based) psychosocial measures, likely to influence an individual psychologically and/or socially [50], including factors related to global/health-related quality of life, and well-being and its derivatives, following an integrated life quality and well-being model [51]. As outcomes, studies had to report PA levels, global and/or discriminated by intensity or domains. Volume, exercise energy expenditure, activity counts, steps, or other measures of PA level were considered. Attendance or compliance with an exercise intervention was not considered as outcome, as this review is focused on adherence to PA in free-living conditions, as part of one’s daily life routines. Lifestyle interventions promoting changes in multiple behaviors (diet + exercise), including pharmacological components, or interventions based on breathing and meditation exercises-only, if not accompanied by real exercise (and thus not contributing to the achievement of PA recommendations), were excluded. Indeed, changes in one health behavior might foster changes into another health behavior [52–54] and confound our results. Pharmacological interventions might interfere with PA participation, depending on its side effects, and were excluded for that reason.

### Search strategy

A comprehensive search of peer-reviewed articles published in English until June 2023 (including online ahead of print publication) was conducted in three electronic databases (PubMed, PsycINFO, and SportDiscus).

Searches included various combinations of three sets of terms: (i) terms concerning the population of interest (e.g., cancer survivors); (ii) terms concerning the intervention(s)/exposure(s) evaluated (e.g., exercise, aerobic or strength training, PA) and the correlates of interest (e.g., psychosocial, cognitive, motivational); (iii) terms respecting the outcomes of interest (i.e., PA adherence, participation, maintenance) (see Additional file 1 for a search example; complete search strategies can be obtained from the authors). Other sources included manual cross-referencing of bibliographies cited in prior reviews [23, 27] and included studies.

### Study selection

All titles and abstracts identified from the literature searches were screened for potential inclusion eligibility by one researcher (EVC). Full texts of potentially relevant papers were retrieved. Three researchers checked if the retrieved papers met the inclusion criteria (BR, JE, SF). Decisions to include or exclude studies in the review were made by consensus. When consensus was not achieved, disagreements were solved by discussion with a fourth author (EVC). The study selection procedure was conducted through the CADIMA software [55].

### Data collection process and data items

A data extraction form was developed, informed by the PRISMA statement for reporting systematic reviews [48]. Data extraction included information about study details (authors, year), participants (characteristics, type of cancer/phase), study design and setting, brief intervention description (including the theoretical framework, if used), intervention and follow-up length, psychosocial correlates (and instruments), PA outcomes of interest (and instruments), and main findings. Two authors (BR, JE) independently coded and extracted the relevant information to be included in the present systematic review. Then, both authors discussed the extracted information with a third author (EVC), deciding what information should be kept by consensus. Data for meta-analysis was also extracted, namely correlation coefficients and sample sizes, or means, standard deviations, and sample sizes per group (active vs. inactive). When data was missing, authors were contacted through email.

### Study quality assessment

Study quality was assessed with an adapted version of the Quality Assessment Tool for Quantitative Studies, developed by the Effective Public Health Practice Project [56]. The current adaptation was based on recommendations from several authors [57–59], and has been previously used [47, 59]. This tool was adapted to allow the evaluation of both experimental and observational studies. It includes 19 items, organized in eight key methodological domains: study design, blinding, representativeness (selection bias), representativeness (withdrawals/dropouts), confounders, data collection, data analysis, and reporting. Each domain is classified as Strong, Moderate, or Weak methodological quality based on specific criteria. A global rating is determined based on the scores of each component. Two researchers independently rated each of the eight domains and overall quality (SF, JE). Rates were discussed by both authors and discrepancies were resolved by consensus. When consensus was not achieved, disagreements were solved by discussion with a third author (BR or EVC).

### Outcome measures

Total PA levels and/or discriminated by intensity or domains constituted the primary outcomes of this review. Volume (minutes per week or day), exercise energy expenditure (Kcal per day or week), activity counts, step counts, or other measure of PA level were considered. Relevant effect measures included simple non-adjusted Pearson or Spearman correlations between psychosocial correlates and PA outcomes, odds ratio, beta regression coefficients, or means (standard deviation) and Cohen’s *d* (standardized mean difference) between active and non-active groups.

### Data synthesis

This review analyzed psychosocial correlates of physical activity in cancer survivors. Characteristics of the included studies were first described by (i) study design, (ii) type and phase of cancer, and (iii) outcomes’ length (short-term as < 6 months or long-term as ≥ 6 months). Then, data were qualitatively synthesized and presented in tabular form. Results are shown separately for each correlate, specifically (i) number of studies, (ii) number of times it was tested (*k*), given that a study could present data for multiple assessment points, (iii) time of outcome assessment—overall; short-term/adherence (< 6 months); long-term/maintenance (≥ 6 months)—, and (iv) the number of times each association effect was found, namely “no association,” “positive association,” or “negative association.” Each correlate was scored as positively or negatively associated if the association was statistically significant (*p* < 0.05); otherwise, no correlate-outcome association was identified. The identified correlates are labelled as reported in the studies. Higher-order categories of correlates were created based on construct conceptual similarity (when justified) to facilitate data synthesis and interpretation.

### Data analysis

Analyses were conducted using the Comprehensive Meta-Analysis (CMA) Software version 3.3.070 [60]. Meta-analyses were conducted for each identified psychosocial correlate, for which there was sufficient data (i.e., when there were 3 or more studies per correlate), to allow interpretability of the data. This option was made given the high variability across studies in design, interventions’ duration and characteristics (when present), sample size, measurement instruments, and the presence of different outcome formats. Meta-analyses were conducted using random-effects models due to the considerable heterogeneity expected among studies.

Effect sizes were computed based on the extracted sample size and simple unadjusted correlation coefficients *r* between each correlate and PA, as reported in the studies or provided by the authors of the paper (which were contacted when these coefficients were not reported in their publications). When studies reported differences in psychosocial correlates between active and inactive groups, the following information was extracted to calculate the effect sizes: (i) mean, SD, and sample size *N*; (ii) *t*-test and sample size *N*; or (iii) standardized mean difference. When data was missing and the information requested was not provided, the study was excluded from the meta-analyses. In the case of studies including different comparison arms (i.e., comparative or controlled trials), discriminated results per groups/arms were preferred. When only results for the whole sample were presented, the decision to include the study in the meta-analysis was made by consensus among the authors, considering the type of psychosocial factor (trait vs. process variable) and the characteristics of the study arms. When similar constructs were measured within the same study and sample (e.g., task self-efficacy and barriers self-efficacy), a combined effect size was estimated to account for the degree of dependence between these measures.

When considered appropriate, based on construct similarity from a conceptual standpoint or original authors’ definition, studies using different measures were included into the same meta-analysis, as if belonging to a higher-order construct (e.g., exercise self-efficacy, barriers self-efficacy, task self-efficacy, and maintenance self-efficacy were grouped into the same higher-order construct—exercise self-efficacy—as their definitions as provided in the papers were very similar). This option was made to allow that effect sizes from more studies could be included in the meta-analyses.

Effect sizes were interpreted according to Cohen’s guidelines [61], with values of 0.10, 0.30, and 0.50 for small, medium, and large effect sizes, respectively. The 95% CI, *Z*-values, and corresponding *p*-values were considered as indicators of the significance of the effect. We also inspected the standard residuals for outliers (> 1.96).

Heterogeneity was tested using the *I*^2^ statistic [62] and the Cochran’s *Q* statistic [63]. The *I*^2^ ranges from 0 to 100%, where a value of 0% indicates no observed heterogeneity and values of 25%, 50%, and 75% reflect low, moderate, and high heterogeneity, respectively [62]. The Cochran’s *Q* statistic demonstrates that studies do not share a common effect size (i.e., there is heterogeneity) when a significant *p*-value (< 0.05) is found [63]. Complementarily, we calculated the prediction intervals, which constitute an index of dispersion, provided in the same units as the effect size, and tell us how much the true effect size varies across populations [64].

The potential for publication bias was subjectively assessed by inspecting funnel plots for asymmetry. They were quantitatively assessed using Egger’s test [65] and Duval and Tweedie’s trim-and-fill method [66] when 10 or more studies were available per meta-analysis and no substantial heterogeneity was present, because the power is too low to distinguish chance from real asymmetry [67].

Sensitivity analyses were carried out to explore whether results were affected by studies with poor quality, or by the different measures grouped together within the same meta-analysis (by construct similarity). Moderator/subgroup analyses were conducted to explore the effect of gender, type and phase of cancer, and outcome’s length, when sufficient data per correlate was available (i.e., at least 2 studies per category).

### Assessment of the certainty of evidence in the present review

Certainty of evidence refers to how confident we can be that a review provides a complete and accurate summary of the best available evidence, and thus, that an estimate of effect is correct [59]. Following the most recent PRISMA recommendations [48], the certainty of the evidence gathered in the present review was assessed with the SURE *checklist* [68]. This checklist includes 5 criteria to assess the identification, selection, and appraisal of studies; 5 criteria to evaluate how findings were analyzed in the review; and 1 criterion for other considerations. Based on the number and type of limitations identified on these criteria, a conclusion regarding the degree of confidence in the evidence of a systematic review is obtained.

## Results

### Search results

**Fig. 1:**
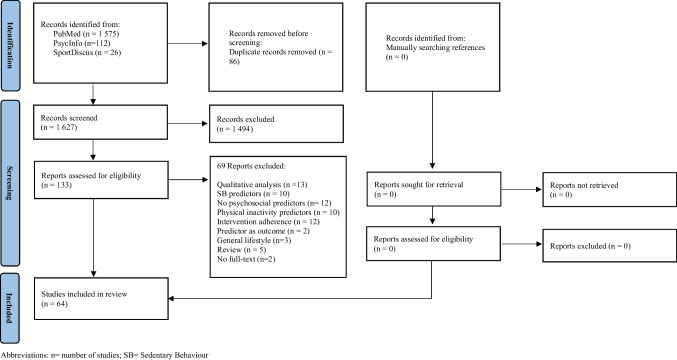
Study selection process flowchart. Abbreviations: n, number of studies; SB, sedentary behavior

Our search resulted in 1713 articles after removing duplicates (86 articles). Based on titles and abstracts, 133 full texts were selected, of which 64 were included in this review. The reasons for exclusion of full texts are described in Fig. 1. Twenty-seven studies were included in meta-analyses [69–86].

### Characterization of studies

Table 1 summarizes the characteristics of all included studies. Overall, most studies were cross-sectional (*n* = 38), three with a prospective design, and 17 studies were randomized controlled trials. The most represented age group was middle adulthood, from 45 to 65 years old (*n* = 41). Most studies included both men and women. However, only four studies focused on men, whereas 23 included women only. Most studies were conducted in the USA (*n* = 25) and Canada (*n* = 20). Four studies were conducted in South Korea, three in Australia, two in Germany, two in France, one in the Netherlands, and another one in New Zealand. Two of the studies had samples from several countries. The most studied type of cancer was breast cancer (*n* = 17), followed by colorectal (*n* = 8) and prostate (*n* = 3). Some studies included multiple cancers and did not specify which were included (*n* = 24).

**Table 1:** Studies characterization

Characteristics	Number of studies	Characteristics	Number of studies
Study design		Outcome assessment length	
Trial		< 6 months	47
Cross-sectional	38	= > 6 months	11
Non-controlled trial	3	Both	6
Non-randomized controlled trial	1		
Cohort observational	2		
Prospective observational	3	Types of cancers	
Randomized controlled trial	17	Bladder	1
		Breast	17
Sample size		Colon	1
< 100	19	Colorectal	8
100–199	16	Endometrial	2
200–299	10	Gynecological	2
≥ 300	19	Head and neck	2
		Kidney	1
Participants		Lung	1
Gender		Lymphoma	1
Both genders	37	Prostate	3
Men only	4	Rectal	1
Women only	23	Multiple cancers	24
Mean age, years			
18–24	1	Quality assessment score	
25–44	3	Weak	38
45–64	42	Moderate	24
≥ 65	12	Strong	2
> 18–75 +	3		
Not reported	3		
Country			
USA	25		
Canada	20		
Brazil	1		
Netherlands	1		
Germany	2		
France	2		
South Korea	4		
Australia	3		
New Zealand	1		
Multiple countries	2		
Sweden	1		
Turkey	1		
China	1		

### Narrative synthesis of results

Additional file 2 describes all results from included articles. A total of 88 different PA correlates (47 theory-based) were tested (Table 2). However, 51% of the correlates were tested less than three times. Self-efficacy was the most tested correlate (*k* = 26), being identified as a positive PA correlate more than half of the times [69, 70, 76, 78–80, 87–101]. Fatigue was the second most frequently tested (*k* = 24) [46, 69–72, 85–88, 90, 93, 101–109], but inconsistent results were found. Enjoyment (*k* = 16) [73–75, 78, 85, 98, 107, 110], perceived behavioral control (*k* = 12) [73–76, 84, 88, 94, 103, 111], intention (*k* = 9) [69, 70, 79, 81, 82, 103, 112, 113], and friend support (*k* = 6) [89, 114–116] were also consistently associated with higher physical activity. Perceived barriers for exercise (*k* = 11) [73–76, 84, 88, 94, 98, 99, 103, 111], perceived social support (*k* = 9) [73, 75, 85, 88, 90, 93, 94, 96, 98], and mental health (*k* = 6) [85, 87, 106, 117, 118] were consistently not identified as a significant correlate. In general, quality of life (*k* = 12) [69, 70, 72, 86, 102, 104, 106, 109, 118, 119], physical functioning (*k* = 10) [46, 69, 77, 86, 105, 106, 109, 118, 119], self-efficacy to overcome exercise barriers (*k* = 8) [73–75, 91, 92, 99, 104, 120], outcome expectations (*k* = 7) [74, 75, 93, 94, 96, 104, 121], and instrumental attitudes (*k* = 6) [69, 70, 79, 82, 112, 113] were positively associated with physical activity, whereas motivation (*k* = 10) [71, 72, 85, 90, 101, 107, 120, 122], subjective norms (*k* = 8) [79, 81, 83, 84, 113, 123, 124], fear/concerns related to exercise (*k* = 7) [71, 72, 75, 84, 125], affective attitudes (*k* = 6) [69, 70, 79, 82, 112, 113], and depression (*k* = 8) [69, 71, 73, 102, 105, 126] showed mixed findings. Several other correlates (78%) were tested five times or less, showing either inconsistent or insufficient results (Table 2).

**Table 2:** Narrative synthesis for physical activity psychosocial correlates

Predictor	Nº studies	Nº times tested	PA outcomes (total)			PA outcomes < 6 m			PA outcomes > 6 m		
			Positive	No association	Negative	Positive	No association	Negative	Positive	No association	Negative
Fatigue	21	24	9	12	3	6	9	1	3	3	2
Exercise self-efficacy	17	26	21	5		12	2		9	3	
Perceived barriers for exercise	13	18	7	9	2	6	6	2	1	3	
Perceived behavioral control	11	12	11	1		9	1		2		
Quality of life	10	12	8	4		4	4		4	1	
PA enjoyment	9	16	12	4		9	4		3		
Perceived social support (general)	9	11	3	8		3	7			1	
Motivation (quantity)	9	10	7	4		4	3		3	1	
Physical functioning	9	10	10			8			2		
Intention	9	9	7	2		5	2		2		
Barriers self-efficacy	8	8	6	2		6	1			1	
Subjective norms	7	8	3	5		3	5				
Depression	7	8	2	5	1	1	4	1	1	1	
Attitude	6	8	2	6		1	5		1	1	
Outcome expectations	6	7	5	2		5	1			1	
Fear/concern related to exercise	5	7		4	3		4	3			
Bodily pain	5	7	1	2	4	1	2	4			
Mental health	5	6		5	1		3	1		2	
Instrumental attitude	5	6	5	1		3			1	1	
Affective attitude	5	5	2	3		2	1			2	
Family support	5	5	1	4		1	4				
Perceived PA benefits	5	5	3	2		3	2				
Social role functioning	5	5		5			5				
Friend support	4	6	5	1		2	1		3		
Physical quality of life	4	5	5			4			1		
Mental quality of life	4	5	2	3		2	2			1	
Injunctive norms	4	4	1	3		1	1			2	
Descriptive norms	4	4	1	3		1	1			2	
Anxiety	4	4		4			1			3	
Positive outcome expectations	4	4	1	3			2		1	1	
Interest in exercise	4	4	3	1		3	1				
Emotional role functioning	4	4	2	2		2	2				
Exercise stage of change	3	8	7	1		4			3	1	
Autonomous motivation	3	4	3	1		2	1		1		
Body image	3	4	1	3		1	3				
General health perceptions	3	4	4			4					
Pros and cons	3	3	1	2		1	1			1	
Perceived stress	3	3		1	2		1	2			
Task self-efficacy	3	3	3			3					
Physical role disability	2	2	1	1		1	1				
Negative outcome expectations	2	2		2			1			1	
Physical well-being	2	2	2			2					
Functional well-being	2	2	1	1		1	1				
Emotional well-being	2	2		2			2				
Difficulty	2	2		2			2				
Social well-being	2	2		2			2				
Happiness	2	2		2			1			1	
Advanced lower extremity function	2	2	2			1			1		
Basic lower extremity function	2	2	1	1			1		1		
Knowledge	2	2		2			2				
Amotivation	2	2			2			2			
Vitality	2	2	2			2					
Cognitive functioning	2	2		2			2				
Family participation	1	6	5	1		3			2	1	
Family reward/punishment	1	6	1	5			3		1	2	
Friend participation	1	6	4	2		2	1		2	1	
Rewards	1	2	2			1			1		
Relatedness	1	2		2			1			1	
Behavior awareness and volition	1	2	2			1			1		
Constructive cognition	1	2	2			1			1		
Symptom burden	1	2		1	1		1	1			
Motivational self-efficacy	1	1		1			1				
Pre-actional self-efficacy	1	1		1			1				
Coping self-efficacy	1	1		1			1				
Relapse self-efficacy	1	1	1			1					
Recovery self-efficacy	1	1		1			1				
Behavioral capability	1	1	1			1					
Doctor encouragement	1	1		1			1				
Self-esteem	1	1	1			1					
Physical health	1	1		1			1				
Role model	1	1		1			1				
PA cons	1	1			1			1			
Outcome expectancies	1	1	1			1					
Controlled motivation	1	1			1			1			
Introjected regulation	1	1	1			1					
Identified regulation	1	1	1			1					
Intrinsic regulation	1	1	1			1					
External regulation	1	1		1			1				
Perceived autonomy support	1	1	1			1					
Competence	1	1	1			1					
Autonomy	1	1	1			1					
Positive affect	1	1	1			1					
Negative affect	1	1			1			1			
Cognitive change processes	1	1		1						1	
Cognitive beliefs	1	1	1			1					
Upper extremity function	1	1		1			1				
Personality	1	1		1			1				
Risk belief	1	1		1			1				
PA facilitators	1	1		1			1				
Perceived susceptibility to cancer recurrence and health problems	1	1		1			1				
Perceived severity of cancer recurrence and health problems	1	1		1			1				
Confidence	1	1		1			1				
Role functioning	1	1	1			1					
Well-being	1	1	1			1					
Lack of interest	1	1			1			1			
Lack of self-discipline	1	1			1			1			

#### Short-term vs. long-term PA correlates

Regarding correlates of short-term PA outcomes (< 6 months), results were similar to the overall results. Exercise self-efficacy [73, 78–80, 88, 91, 99–101], perceived behavioral control [79, 81–84, 88, 112, 113, 123, 124], intention [69, 79, 81, 82, 112, 113], enjoyment [73–75, 78, 85, 107, 110, 127], and outcome expectations [74, 75, 93, 94, 96, 104] generally showed consistent associations with higher physical activity levels. Inconsistent findings were observed for fatigue [46, 71, 72, 85, 86, 93, 101–109, 127], perceived barriers for exercise (reversed) [73–75, 84, 94, 99, 103, 111, 127], subjective norms [79, 81, 83, 113, 123, 124], depression [71, 73, 102, 105, 126], fear/concerns related to exercise [71, 72, 75, 84, 125], and motivation (quantity) [71, 72, 85, 90, 101, 107, 120, 122]. Social support [73, 75, 88, 93, 94, 96, 107, 119] was consistently identified as a non-significant correlate. Long-term PA correlates (≥ 6 months) were tested much fewer times, though generally reproducing the trends observed for the overall and short-term PA outcomes, especially for exercise self-efficacy (positive correlate) and fatigue (inconsistent correlate). Quality of life and motivation (quantity) apparently show more consistent and positive associations in the long-term but were studied only 4–5 times. All other variables were insufficiently investigated.

#### PA correlates per type of cancer

Concerning cancer type, for breast cancer, 32 different PA correlates were tested, but only 11 more than three times (Additional file 3). Exercise self-efficacy was identified as a positive correlate of physical activity in most studies, including in the long-term. Fatigue, perceived barriers for exercise, outcome expectations, and perceived social support showed mixed findings. Other variables were insufficiently studied to allow solid conclusions, but in some cases are suggestive of a positive association with physical activity, namely self-efficacy to overcome exercise barriers, quality of life, PA enjoyment, friend support, perceived behavioral control, and physical functioning. For colorectal cancer (Additional file 4), 42 different potential correlates were tested, but only one more than three times. Evidence is mixed for the association between fatigue and overall and short-term PA outcomes. No sufficient data was available for the remaining variables.

### Meta-analytic results

Twenty-three psychosocial correlates were tested in meta-analyses (Table 3). Of these, all but five (i.e., fatigue, motivation, social support, anxiety, and bodily pain) were identified as significant correlates. Moderate magnitude pooled effect sizes were found for exercise self-efficacy (*n* = 11; *r* = 0.38; 95% CI 0.30, 0.46), perceived behavioral control (*n* = 9; *r* = 0.34; 95% CI 0.28, 0.40), intention to be physically active (*n* = 7; *r* = 0.45; 95% CI 0.31, 0.56), perceived barriers for exercise (*n* = 6; *r* =  − 0.34; 95% CI − 0.44, − 0.23), enjoyment (*n* = 6; *r* = 0.35; 95% CI 0.27, 0.43), and perceived benefit of PA (*n* = 3; *r* = 0.36; 95% CI 0.26, 0.45), although high heterogeneity (*I*^2^ ~ 90%; 95% PI − 0.07, 0.77) was observed for intention and results were based on few studies for perceived PA benefits (less than 5). A moderate-magnitude positive effect size was found for global attitudes (*n* = 8; *r* = 0.32; 95% CI 0.24, 0.40). Additional meta-analyses for the two components of attitudes were conducted, showing similar results (affective: *n* = 5; *r* = 0.30; 95% CI 0.20, 0.40; instrumental: *n* = 5; *r* = 0.28; 95% CI 0.17, 0.39), though with substantial heterogeneity (*I*^2^ > 75%; 95% PI crossing zero, suggesting a high dispersion of effects, including in the opposite direction). A small to moderate positive effect size was found for subjective norms (*n* = 8; *r* = 0.22; 95% CI 0.11, 0.32), but with high dispersion of effects and potential effects in the opposite direction (95% PI − 0.13, 0.52). In addition, when looking into the results from the additional meta-analysis performed for each component of subjective norms, small to negligible effects were observed (injunctive: *n* = 4; *r* = 0.13; 95% CI 0.04, 0.23; descriptive: *n* = 4; *r* = 0.08; 95% CI 0.02, 0.13). Small to moderate effect sizes were also found for physical functioning (*n* = 9; *r* = 0.23; 95% CI 0.17, 0.29), quality of life (*n* = 7; *r* = 0.18; 95% CI 0.13, 0.22), depression (*n* = 4; *r* =  − 0.21; 95% CI − 0.40, − 0.01), and mental health (*n* = 3; *r* = 0.21; 95% CI 0.12, 0.29), although based on few studies (less than 5) for the latter two correlates, and high dispersion of effects for depression and potential effects in the opposite direction (95% PI − 0.81, 0.61). In general, these findings suggest that higher levels in most of these factors (lower for perceived barriers and depression) are associated with higher PA levels. Publication bias could not be tested for most variables as there were less than 10 studies per correlate (note: there was no publication bias for physical activity self-efficacy, based on the visual inspection of the funnel plot (see Additional file 5) and Egger’s test (*p* > 0,05) [58]. Moderator/subgroup analyses exploring the effect of study design, gender, type and phase of cancer, or outcome’s length could not be performed, as no sufficient data was available.

**Table 3:** Meta-analyses results for physical activity psychosocial correlates

	Meta-analyses					
PA correlates	*r*	95% CI	*Z*	*Q*	*I*^2^	95% PI
Exercise self-efficacy (*n* = 11)	0.38	0.30, 0.46	8.36***	25.76**	61.2	0.11,0.60
Perceived behavioral control (*n* = 9)	0.34	0.28, 0.40	9.72***	16.5*	51.5	0.15, 0.51
Fatigue (*n* = 9)	− 0.07	− 0.23, 0.11	− 0.73	81.3***	90.2	− 0.60, 0.50
Physical functioning (*n* = 9)	0.23	0.17, 0.29	7.60***	17.5	54.4	0.07, 0.38
Subjective norms (*n* = 8)	0.22	0.11, 0.32	3.97***	32.3***	78.3	− 0.13, 0.52
Attitudes (*n* = 8)	0.32	0.24, 0.40	7.16***	19.4**	63.9	0.06, 0.54
Intention (*n* = 7)	0.45	0.31, 0.56	5.91***	55.5***	89.2	− 0.07, 0.77
Quality of life (*n* = 7)	0.18	0.13, 0.22	7.28***	8.86	32.3	0.07, 0.27
Perceived barriers for Exercise (*n* = 6)	− 0.34	− 0.44, − 0.23	− 5.95***	7.01	28.7	− 0.55, − 0.08
Enjoyment (*n* = 6)	0.35	0.27, 0.43	7.96***	6.18	19.1	0.17, 0.51
Depression (*n* = 4)	− 0.21	− 0.40, − 0.01	− 2.1*	18.8***	84.1	− 0.81, 0.61
Social support (*n* = 4)	0.12	− 0.01, 0.25	1.77	2.04	0.00	- ^a^
Motivation (*n* = 4)	0.17	− 0.16, 0.46	0.99	16.0**	81.3	− 0.85, 0.92
Mental health (*n* = 3)	0.21	0.12, 0.29	4.45***	1.46	0.00	- ^a^
Anxiety (*n* = 3)	0.03	− 0.07, 0.13	0.65	0.65	0.00	- ^a^
Bodily pain (*n* = 3)	0.05	− 0.29, 0.38	0.29	17.6***	88.6	− 1.00, 1.00
Benefit of PA (*n* = 3)	0.36	0.26, 0.45	6.95***	1.29	0.00	- ^a^

^*^ < 0.05; ** < 0.01; *** < 0.001; *95% CI*, 95% confidence intervals; *Q*, Cochran’s *Q* statistic, measure of heterogeneity; *I*^2^ statistic, measure of heterogeneity; *95% PI*, 95% prediction intervals indicate dispersion of effect sizes and are a measure of heterogeneity; ^a^Prediction interval could not be calculated, given that tau-squared was 0.000[64]

### Quality assessment

Regarding the overall methodological quality of the studies (Table 1), only 2 studies were rated as “strong,” 24 studies were classified as “moderate,” and 38 were rated as “weak.” The main limitations detected concern the study design (most studies were observational), absence of adequate blinding (for experimental designs), representativeness (all, but two, were composed of volunteers and not representative), and lack of adequate adjustment of analysis for confounders. Quality assessment results for each study can be found in Additional file 6.

### Assessment of the certainty of evidence in the present review

The SURE checklist (Additional file 7) indicated this is a good systematic quality review, with only minor limitations: language bias was not avoided, given that only papers in English were included. A more comprehensive search could have resulted in a higher number of retrieved papers. Even taking these results in consideration, the findings of the current systematic review can be considered as reliable, although based on a limited number of studies per outcome. Hence, further research is required to confirm these findings.

## Discussion

The current review proposed to identify psychosocial correlates of free-living PA in cancer survivors. Eighty-eight potential correlates of free-living PA were tested. Most variables (78%) were tested less than 6 times, showing either inconsistent or insufficient results. In global terms, exercise self-efficacy, perceived behavioral control, intention, quality of life, outcome expectations, instrumental attitudes, friend support, enjoyment, and physical functioning were positively associated with physical activity, whereas perceived barriers for exercise revealed negative associations with physical activity. Perceived social support and mental health were consistently identified as a non-significant correlate. Fatigue, subjective norms, fear/concerns related to exercise, affective attitudes, motivation (quantity), and depression showed mixed findings. These trends were similar for short-term PA outcomes and for long-term PA outcomes (though these were far less tested).

Meta-analyses were performed for 21 correlates (i.e., the ones tested more than 3 times), of which 16 showed significant pooled effects. Results were generally in line with the narrative findings, with a few exceptions, namely for subjective norms, attitudes, and depression, which revealed significant pooled effect sizes (positive for the first two; negative for the latter), possibly due to the lower number of studies providing data to be included in the meta-analyses. Moderate magnitude effect sizes were observed for exercise self-efficacy, perceived behavioral control, intention, perceived barriers for exercise, enjoyment, perceived PA benefits, and attitudes (globally and both dimensions). Small to moderate effect sizes were found for subjective norms (but not for its dimensions), physical functioning, quality of life, depression, and mental health, though based on less than 5 studies for the latter two correlates. In general, these findings suggest that higher levels in most of these factors (lower for perceived barriers and depression) are associated with higher PA levels.

Exercise self-efficacy was the most consistent positive correlate of free-living physical activity. Indeed, self-efficacy is included in several other theories besides Bandura’s socio-cognitive model [128], which may explain its extensive testing. This finding reinforces the relevance of this construct in the psychological dynamics underlying PA participation [129, 130]. Hence, future studies in this population should devote to the testing of the most effective strategies to increase self-efficacy, aiming at more effective interventions in this regard.

We also found positive associations with perceived health (physical functioning) and quality of life (physical component, and general quality of life). In many studies, these variables are considered outcomes, but in the present review they were included as correlates, similarly to other reviews [23, 131], given that a bidirectional effect may apply: besides being possible correlates of free-living PA adoption, they are also outcomes directly affected by PA [12]. In fact, it is plausible that it is PA participation that is indeed reinforcing these variables and not the other way around [132, 133]. Previous reviews [23, 27] did not include observational studies, and the outcomes used as inclusion criteria were slightly different from ours, as the focus was exclusively on PA intervention compliance/adherence and not on PA participation in free living conditions.

Inconsistent results were found for fatigue, which has been previously reported as an important barrier [25, 26]. This inconsistency may be due to the use of different scales to measure perceived fatigue, or inherent differences across cancer types, cancer stages, and treatment processes, or even to patients’ beliefs and expectations. More longitudinal data exploring the role of this key construct, using standardized and cancer-specific scales, across cancer types and survivorship stages is in need, given its bidirectional function as either PA correlate or outcome in this specific population of cancer survivors [12]. Also, it would be relevant to explore whether certain characteristics like treatment types, time since diagnosis, medication, or the presence of comorbidities moderate the associations between perceived fatigue and PA.

Results for enjoyment (a central marker of intrinsic motivation) warrant further reflection. Contrary to the evidence on healthy populations [134, 135], enjoyment was not consistently associated with PA in the narrative synthesis (a positive association was found in half of the times it was tested; and no association in the other half), although the meta-analysis revealed a significant, moderate, positive pooled effect size. It is important to note that all the studies testing this correlate were in the short-term, and this may be a more central correlate for the maintenance of the behavior. Furthermore, the mixed findings concerning enjoyment might not be that surprising in cancer populations (vs. healthy populations): Given that the review has included patients diagnosed with diverse cancer types, in several phases of treatment, and going through different treatments (some more aggressive than others, thus more draining to the individual), a different dynamic might be expected. Future research would do well to explore these aspects as potential moderating factors, capable of explaining why enjoyment might be a correlate of PA for some patients, but not for others. In addition, most of the correlates identified in this systematic review pertain to cognitive domains (self-efficacy, intention, outcome expectations, perceived behavioral control, and instrumental attitudes) and not so much to affective domains, which is consistent with previous research with other types of debilitating conditions, such as fibromyalgia [130], obesity [47], multiple sclerosis [136], or individuals with disabilities [137].Thus, this finding may need further testing in trials to come, as it might have central implications in the way PA is promoted among cancer survivors.

Attitudes (general), subjective norms, and perceived social support tended to be unrelated or inconsistently related with PA among cancer survivors, which is a remarkable difference from results obtained in healthy populations [129, 138]. Interestingly, significant positive associations between attitudes/subjective norms and PA were observed in meta-analyses. This inconsistency could be explained by the limited number of studies included in each of these meta-analyses, which might not entirely represent the overall results found in the literature. There is a clear need for better quality studies, that properly address these associations and thus provide quantitative data to confirm or refute these findings. It is also worth to mention that in our meta-analyses, descriptive norms (i.e., perceptions of which behaviors are typically performed) had a negligible positive association with PA, while injunctive norms (i.e., perceptions of which behaviors are typically approved or disapproved in society) revealed a small-magnitude positive association. Although this finding requires further confirmation given it is based on a limited number of studies, it may suggest that for cancer survivors the cultural and community acceptability and social (perhaps even medical) approval of PA behaviors might be more important than the perception of other’s PA participation.

Overall, the more consistent correlates of free-living PA identified in the present systematic review were derived from theoretical models, further supporting the relevance of designing and implementing theory-based interventions to promote physical activity, as previously recommended [139–141]. The identified theoretical correlates came mainly from SCT (e.g., self-efficacy, outcome expectations, perceived barriers) and TPB (e.g., perceived behavioral control, intention, attitudes, subjective norms), in line with findings from other reviews [23, 27]. Nonetheless, superiority of these theories in the prediction of PA in this population cannot be implied. Indeed, it is necessary to keep in mind that these were also the most tested theories, which results in a greater exploration of these constructs compared to other theory-related constructs. For example, exercise stages of change from TTM have been identified as a significant correlate in a prior review in cancer survivors [27]; however, this construct was tested only twice in the present review precluding solid conclusions. Furthermore, this may be explained by the fact that these constructs are most used to predict adherence to PA interventions and not to PA in free-living conditions.

Of note, SDT-based constructs (e.g., need satisfaction, autonomous motivations, enjoyment) have been consistently showing favorable effects on PA adherence in several other populations [42], but remain poorly tested in cancer populations. Furthermore, when motivation is tested in this population, it is reported using different scores/variables (e.g., total score of motivation reflecting only a quantitative perspective on motivation) than the ones embedded in SDT. Nevertheless, taken together, results from SDT constructs in our review seem to follow the same trend observed for other healthy and clinical populations, suggesting that more self-determined (autonomous) motivations might be a positive correlate of free-living PA in cancer survivors as well.

This review also showed similar trends for short- and long-term PA psychosocial correlates. However, for more consistent results in the long-term, more studies are needed. Follow-ups longer than 6 months were uncommon among the included studies, which suggests that long-term PA correlates remain poorly tested, as previously stated [28]. Curiously, among the few studies reporting this data, findings suggest a positive association between friend support (but not with family support) and free-living PA, especially in the long-term. This suggests that social support may have different facets that should be personalized to the survivors’ needs and preferences. In addition, this factor might be more relevant for the maintenance rather than initiation of PA amid cancer survivors. As an additional note, the increasing interest in the Health Action Process Approach–related constructs such as social support may reveal an attempt from researchers to understand and document behavior maintenance processes. Indeed, past research has noted the importance of the Health Action Process Approach model for the understanding of PA behavior [142–144].

Using a “one size fits all” approach to promote sustained PA adherence has been previously proven ineffective [145]. However, although it may be useful to know whether the type of cancer has any influence in the role of each psychosocial factor on free-living PA, this review clearly showed that robust data per type of cancer was lacking, even for the two most studied types of cancer, breast and colorectal. There is still insufficient data to allow withdrawing conclusions regarding the most relevant PA correlates per type of cancer. Having this information would facilitate the development of tailored interventions, possibly leading to more successful outcomes.

PA is being increasingly integrated as part of care for cancer survivors [9–12] due to its potential for disease and treatment management, as well as for survivors’ health improvement [4–8]. Thus, the identification of significant psychosocial correlates of free-living PA may be of crucial importance to best inform the development of PA promotion interventions and, subsequently, increase cancer survivors’ participation in physical activities.

### Limitations and strengths

This systematic review sought to comprehensively identify the most relevant psychosocial correlates of PA among cancer survivors, including experimental but also observational study designs, and by putting the focus on physical activity in free-living conditions, and not on PA session attendance or intervention compliance. This focus is essential for future research and practice because the ultimate challenge is long-term integration of PA in cancer survivors’ daily life.

This review has also some caveats that need to be discussed. First, due to a small number of studies per correlate, meta-analyses could only be performed for 21 correlates among the identified 83. Second, we could not perform subgroup analysis, concerning the time length of follow-ups, cancer type, cancer stage, gender, or study design, given the scarcity of studies per correlate. This would be of relevance given the high heterogeneity observed for some of the correlates. Third, most of the included studies were correlational in nature and had a poor methodological quality overall, suggesting the need for improvements in research methodology, especially at the level of selection bias, blinding, and adjustment for confounders. Fourth, the screening of titles/abstracts was performed by a single author, which could have led to the exclusion of relevant studies. However, all doubts regarding the inclusion of studies were discussed with the other authors and decisions were made by consensus.

## Conclusion

The current systematic review highlights key psychosocial correlates of free-living PA adherence, which are of fundamental interest to inform future public health interventions and policies related to PA promotion among cancer survivors. Constructs mainly from socio-cognitive theory and the theory of planned behavior were consistently associated with free-living PA among cancer survivors. However, at the present time, we cannot assert which frameworks might be more effective, nor whether other promising, but insufficiently studied, theoretical constructs might play a greater role in the prediction of free-living PA adherence. Finally, further studies of better methodological quality, per correlate, exploring longer-term associations with PA and across different types of cancer, are needed to confirm and/or extend the results of the present review. A greater standardization of methods and instruments to assess PA and psychosocial correlates should be sought out to allow more robust and insightful conclusions.

## Supplementary Information

Below is the link to the electronic supplementary material.

Supplementary file1 (DOCX 13 KB)

Supplementary file2 (DOCX 60 KB)

Supplementary file3 (DOCX 21 KB)

Supplementary file4 (DOCX 23 KB)

Supplementary file5 (DOCX 106 KB)

Supplementary file6 (DOCX 35 KB)

Supplementary file7 (DOCX 16 KB)

## Data Availability

No datasets were generated or analysed during the current study.

## Declarations

### Registration and protocol

This review was registered on the PROSPERO database (CRD42021259244). The review protocol was not published.
