# Supplementary material for: Psychosocial correlates of physical activity in cancer survivors: a systematic review and meta-analysis
Source: J Cancer Surviv. 2024 Mar 6;19(4):1385–402. doi: 10.1007/s11764-024-01559-6 (PMC12283835; doi:10.1007/s11764-024-01559-6)
Supplement: Supplementary file 1 — Supplementary file1 (DOCX 13 KB) [file 11764_2024_1559_MOESM1_ESM.docx]

**Additional file 1 - Search Strategy**

**PubMed, PsycINFO, and SportDiscus**

(cancer survivors OR cancer patients)

AND

(physical activity OR exercise OR aerobic exercise OR strength training OR resistance training OR walking OR running OR jogging OR swimming OR dance)

AND

(participation OR adherence OR maintenance OR adoption)

AND

(correlates OR determinants OR predictors OR barriers OR factors OR facilitators OR motivators)

AND

(psycho* OR social OR affective OR cognitive OR social-cognitive OR motiv* OR self-regulat* OR aspirat* OR expectat* OR self-determin*)
