# Supplementary material for: Psychosocial correlates of physical activity in cancer survivors: a systematic review and meta-analysis
Source: J Cancer Surviv. 2024 Mar 6;19(4):1385–402. doi: 10.1007/s11764-024-01559-6 (PMC12283835; doi:10.1007/s11764-024-01559-6)
Supplement: Supplementary file 2 — Supplementary file2 (DOCX 60 KB) [file 11764_2024_1559_MOESM2_ESM.docx]

**Additional File 2 - Results**

Table of results

| **Authors**  **(Country)** | **Study Design** | **Cancer Type, Phase** | **Sample (n; % women; Age mean ± SD or Age range (years); BMI** | **Intervention (or exposure)** | **Psychosocial Predictors** | **PA Outcomes** | **Results** |
| --- | --- | --- | --- | --- | --- | --- | --- |
| Bennett et al., 2007  (USA) | RCT | Multiple cancers, who completed  treatment at least 6 months prior to enrolment | 56; IG: 28; 93% women; 55.5 ± 8.9 years; 29.6 ± 6.4 kg/m2.  CG: 28; 86% women; 26.6 ± 7.2 kg/m2 | The intervention consisted of one in-person counselling session followed by two telephone calls over 6 months. | Self-efficacy for exercise (set of items);  Stage of Change (PASA-NIH);  Fatigue (SCFS);  Mental Health Status (MOS SF-36) | Caloric expenditure (kcal/week; CHAMPS) | High-efficacy individuals in the intervention group increased their PA levels faster over 6 months than did low-efficacy individuals in the intervention group.   Fatigue, mental health and stage of change were not significant. |
| Courneya et al., 2012  (Canada) | RCT | Lymphoma, receiving chemotherapy or no treatments | 110; 43.6% women; 53 years; N.R. | IG: Perform supervised exercise on an upright or recumbent exercise bike three times per week for 12 weeks at a university-based fitness centre.   CG: participants were  asked not to increase their exercise during the intervention | Quality of life (FACT-An and lymphoma subscale);  Physical functioning (TOI-An);  Fatigue (Fatigue subscale from the FACT-An);  Happiness (Happiness Scale);  Depression (CES-D short form);  Anxiety (SSAI short form);  Intention, perceived control, self-efficacy, affective attitude, instrumental attitude, injunctive norm, descriptive norm (based on a standard format recommended by Ajzen). | Exercise behaviour at 6-month follow-up (min/week; modified version of GLTEQ) | Not meeting exercise guidelines (n=49; 45.4%) VS. Meeting exercise guidelines (n=59; 54.6%)  Meeting guidelines are associated with post-intervention intention, change in intention, post-intervention perceived control, change in perceived control, post-intervention self-efficacy, post-intervention physical functioning, Post-intervention QoL, post-intervention fatigue, post-intervention depression.  No associations in change in self-efficacy, post-intervention affective attitude, change in affective attitude, post-intervention instrumental attitude, change in instrumental attitude, post-intervention injunctive norm, change in injunctive norm, post-intervention descriptive norm, change in descriptive norm, change in physical functioning, change in QoL, post-intervention happiness, change in happiness, Change in fatigue, post-intervention anxiety, change in anxiety, change in depression |
| Courneya et al., 2004  (Canada) | RCT | Colorectal, had surgery for colorectal cancer within the past 3 months | 93; 61.9 % woman; 60.3 ± 10.4 years; N.R. | IG: home-based, personalized exercise program. The  goal was to have participants exercising at least 3–5 times per week, for 20–30 min, at 65–75% of the predicted heart rate maximum  CG: were asked not to begin a structured exercise program and were not given an exercise prescription. | Exercise stage of change (set of items)  TPB constructs (Attitude, PBC; Subjective norm; set of items)  Personality (NEO 4FI)) | Exercise (LSI- GLTEQ) | For the exercise group, was found a positive significant association between exercise adherence and exercise stage and perceived behaviour control .  Attitude, personality, and subjective norm was not associated.  Independent predictors of exercise adherence in the final equation were exercise stage, and PBC |
| Hiensch et al., 2020  (Netherlands) | RCT | Breast and Colon cancer, Stage M0 Scheduled for chemotherapy | Overall: 237; 55.8 ± 8.2 years; 92% women; 26 ± 4.1 kg/m2  IG: n=119 CG: n=118 | IG: 18-week supervised aerobic and muscle strength exercise program in addition to the usual care.  CG: usual care and were asked to maintain their habitual PA pattern up to week 18. | Fatigue (MDI)  HRQoL (EORTC-QOL-C30);  Anxiety and Depression (Hospital Anxiety and Depression Scale). | PA levels 4-years after participation in the PACT study (SQUASH): Minutes per week of MVPA and leisure and sport activity  0, 4 years | Lower baseline physical fatigue was significantly associated with total PA, and leisure and sport activity.  Lower physical fatigue and having a positive change in physical fatigue during the intervention period were correlates of sport and leisure PA levels 4 years post-baseline.  HRQoL, anxiety and depression were not associated with PA. |
| James et al., 2006  (USA) | RCT | Colorectal  cancer | 304; 46.1% women; 65.2 ± 10.6 years; N.R. | 2x2 factorial intervention design, testing 2 different methods of communicating and promoting health behavior change (tailored print messages and telephone-based motivational interviewing) | Self-efficacy (1 item);  Perceived social support (set of items);  Perceived barriers (set of items);  Knowledge of recommendations (set of items) | PA (min/week; modified  version 7-day PA recall)  Baseline, with follow-up at 6- and 12-  months. | Self-efficacy was positively associated with PA.  Barriers, perceived social support and knowledge of recommendations were not associated with PA. |
| Kwarteng et al., 2020  (USA) | RCT | Breast cancer, Stage I-III, Completed treatment > 6 months | Overall: 246 women; 57.5 ± 10.1 years; 36.1 ± 6.2 kg/m2  IG: 125 women; 56.8 ± 10.0 years; 35.9 ± 6.2 kg/m2 CG: 121; 58.1 ± 10.1 years; 36.4 ± 6.4 kg/m2 | IG: Twice-weekly in-person classes with supervised exercise and twice-weekly text messaging targeting enhanced self-efficacy, social support and access to health promotion resources.  CG: Self-guided weight loss. No exercise and no text messages. | Social support from family and friends for exercise (SSQ);  Self-efficacy (SESWL). | Leisure time PA in MET-hours per week (MAQ).   6 months of interventions and follow-up at 12 months. | Self-efficacy and friend support for exercise habits-participation were positively associated with increased minutes per week of MVPA in the IG (vs.CG), at 6 and 12 months.  Family support was not associated with PA. |
| Lee et al., 2016  (Canada) | Single group intervention | Breast cancer, stage 0–III who completed chemotherapy and/ or radiation therapy. | 42 women; 53.8 ± 9.2 years; 31.2 ± 6.3 kg/m2 | IG; Six-week PA intervention focuses on improving PA self-efficacy and outcome expectations so as to enhance PA behaviour. Six weekly 2.5-hour structured classes consisted of education and  practice sessions on aerobic, resistance, balance, and  flexibility exercises that could be performed at home. | Fatigue (FACT-F)  PA self-efficacy (PASES) | PA level 6 months post intervention (min/week; interviewer-based 7-Day PA Recall) | The first model included only baseline fatigue as a significant factor: baseline fatigue accounted for only 12% of the variance of PA maintenance.  Self-efficacy was not associated. |
| Mama et al., 2017  (USA) | RCT | Breast cancer, stages I–IV, at least 3 months post-treatment | 89 women; 58.5 ± 9.2 years; 31.0 ± 6.5 kg/m2 | IG: completed a 16-week culturally adapted and standard exercise intervention that targeted aerobic exercise,  muscular strength, and flexibility training, and received biweekly phone calls to check on their progress and biweekly newsletters.   Other groups: a non-culturally adapted standard exercise intervention, or a wait-list control treatment group. | Exercise self-efficacy (set of items);  Barriers self-efficacy (CSEBSES); Social modeling of PA (set of items); | PA (hours and/or minutes per day and days per week and yielded total minutes of PA per week) and walking at 16-week; IPAQ short form). | Increases in barrier self-efficacy were positively associated with reported increases in walking in the intervention group, whereas women in the control group reported only slight increases in walking from baseline to 16-week post-intervention.   In unadjusted models, changes in exercise self-efficacy were significantly associated with changes in walking, and this association remained significant after adjusting for age, site, and treatment group, suggesting that increases in exercise self-efficacy were associated with increases in walking from baseline to follow-up.  No significant association for MVPA and total PA |
| Mayer et al., 2018  (USA) | RCT | Colon Cancer, Stage I-III, Completed cancer treatment and at least 6 weeks postoperative to within 12 months of the diagnosis with no sign of recurrence | Overall: 284; N.R.; N.R.; N.R.;  IG: 144; 51% women, 57.84 ± 14.5 years; N.R.  CG:140; 52% women; 59.34 ± 13.7 years; N.R. | IG: all usual care plus smartphones with the SurvivorCHESS application, along with voice and data services for the study period.  CG: Usual care: National Cancer Institute’s Facing Forward: Life after Cancer Treatment booklet, the National Coalition for Cancer Survivorship’s Cancer Survival Toolbox, and a pedometer. | Autonomy and relatedness (TSRQ). | PA (GLTEQ): Times of 15min-PA per week.  0, 3, 6, and 9 months. | Relatedness was not a significant predictor of PA, controlling for the intervention group.  Autonomous motivation was not found to mediate the effect of the SurvivorCHESS intervention on physical activity: the intervention did not significantly predict autonomous motivation at three months, though autonomous motivation at three months was significantly predictive of physical activity at six months when controlling for intervention group. |
| Mazzoni et al., 2021 | RCT | patients newly diagnosed with breast,  colorectal or prostate cancer | Overall 301  BCT HI=77; 79% women; 60 ± 12 years; BMI 26 ± 4  BCT LMI =81; 79% women; 58 ± 12 years; BMI 25 ± 4  HI=71; 79% women; 57 ± 11 years; BMI 25 ± 4  LMI=72; 79% women; 60 ± 11 years; BMI 25 ± 4 | Participants exercised for six  months while undergoing cancer treatment. The exercise programme consisted of supervised group-based resistance training and individual home-based endurance training.  Participants alternated between 3×6 repetitions maximum (RM) and 3×10 RM in the HI groups, and 3×12 repetitions at 50% of 6 RM and 3×20 repetitions at 50% of 10 RM in the LMI groups. The endurance training consisted  of twice-weekly interval training (20–40min/session) at  80–90% of heart rate reserve (HRR) in the HI groups, and  150 min weekly continuous-based exercise at 40–50% of  HRR in the LMI groups.  BCT: use goal-setting, review of behavioural goal, self-monitoring, action planning and problem solving. | Anxiety  Cancer-related fatigue  HRQoL  Exercise self-efficacy  Exercise expectations  Exercise motivation | A tri-axial PA  monitor, the SenseWear Armband mini (SWA) [28, 29]  and a 7-day PA diary  0, 6, 12 months | We found that self-regulatory BCTs improved PA maintenance at 12-month follow-up in our study sample, especially in participants exercising at high intensity during the intervention. Further, higher HRQoL and higher exercise motivation at baseline were positively associated with PA maintenance at 12-month follow-up. In contrast, higher exercise expectations, being former or current smokers/snus users at baseline and gaining weight during the exercise intervention were negatively correlated with PA maintenance at 12-month follow-up. Cancer-related fatigue and exercise self-efficacy were not associated.  .  Twelve months after the Phys-Can RCT, the odds of maintaining PA among participants receiving self-reg-  ulatory BCTs were 1.8 times the odds of maintaining PA among those who did not receive self-regulatory BCTs. |
| Mosher et al., 2013  (USA) | RCT | Breast and prostate cancer, early stage (in situ, localized, or regional) | 237 for breast, and 252 for prostate; 57.2 ± 10.7 years; N.R. | IG: For 10 months participants received an initial workbook followed by a series of seven newsletters at 6-week intervals. Newsletters were tailored to the experimental participants’  demographic characteristics (age, race, and sex), cancer coping style, stage of readiness, barriers to health behaviour change, and progress toward goal  behaviours (150+ minutes of PA, adherence to a low fat or high F&V diet)  CG: received an initial workbook that included the ‘Facing Forward’ booklet from the National  Cancer Institute and subsequent publicly available materials  on PA, F&V intake, and dietary fat restriction | Self-efficacy (single item)  Barriers (set of items) | PA at 2 years of follow up (min/week MVPA; 7-day PA Recall). | Change in self-efficacy for PA was positively correlated with total minutes of PA per week, and change in barriers to PA was negatively correlated with PA. |
| Pinto et al., 2023  (USA) | RCT | Diagnosed in the past five years with Stage 0–3 breast cancer | 161 women,  57.33y ± 10.85 | All three study groups were structurally equivalent with  respect to the 3-month PA intervention (12-weekly calls  from coaches, PA logs, pedometer and feedback reports).  a) Reach Plus & maintenance program (In Months 1–3, participants in this group  received the previously tested telephone counselling for PA; Months 4-9: participants were provided PA logs for the remaining months. They were encouraged to continue to use the heart rate monitors and pedometers during PA and mail/e-mail the logs.  b) Reach Plus Phone (after the 3 M assessments were completed, the coaches were asked to continue to contact their participants each month during Months 4–9).  c) Reach Plus Message (participants received brief messages once per week by email or text (as determined by participants’ preferences) to motivate, prompt and reinforce continued PA. | Stage of Readiness for Exercise (Marcus et al., 1992)  Exercise Self-Efficacy (5-item Exercise Self-Efficacy measure)  Social Support (Social Support for Exercise Survey)  Physical Activity Enjoyment (18-item Physical Activity Enjoyment Scale)  Exercise Barriers (Sechrist et al., 1987) | Actigraph (Model  GT3X) accelerometer  Seven Day Physical Activity Recall (7 Day PAR)  6, 9, 12 months | Self-efficacy was positively correlated with the 3 interventions at 3M and with 2 interventions at 6M. No correlation for 1 intervention at 6M.  Family participation was positively correlated with the 3 interventions at 3M and with 2 interventions at 6M. No correlation for 1 intervention at 6M.  Family rewards/punishment was positively correlated with the 1 intervention at 6M. No correlation for 3 interventions at 3M and 2 interventions at 6M.  Friend participation was positively correlated with 2 interventions at 3M and 6M. No correlation for 1 intervention at 3M and 6M.  Enjoyment was positively correlated with all 3 interventions at 3M and 6M.  Exercise barriers were not correlated with all 3 interventions at 3M and 6M.  Exercise stage of change was positively correlated with all 3 interventions at 3M and 6M. |
| Papadopoulos1 et al., 2022  (Canada) | RCT | Prostate cancer survivors | 37 men; 69.4 ± 6.5 years; 28.2 ± 24.4-30.8 kg/m2 | 6-month exercise intervention via personal training (PT), supervised  group-based (GROUP) training, or home-based (HOME)  training.  4–5 days per week of mixed modality exercise, incorporating aerobic, resistance, and flexibility training. The target time and relative workload (target heart rate 60–70% of heart rate reserve)  were consistent across all intervention groups. | Planning, Attitudes, & Barriers  (PAB) Scale | - Baseline and follow-up 6M  Self-reported PA (Godin Leisure-Time Exercise  Questionnaire (GLTEQ))  Objectively measured PA (Actigraph GT3X) | Attitude (r=0.64; p<0.01), and motivation (r=0.50; p<0.05) were positively correlated with higher MVPA levels from baseline to 6 months, measured through accelerometry (n=14).  Attitude (r=0.28; p<0.10), and motivation (r=0.1) show no correlation for MVPA changes measured with GLTEQ (n=37). |
| Rogers et al., 2011  (USA) | RCT | Breast cancer, Stage I, II, or IIIA breast cancer currently being treated with an aromatase inhibitor or estrogen receptor modulator | 36 women; 53 ± 9 years; N.R. | The 3-month intervention included 12 individual supervised exercise sessions, 6 discussion group sessions, and 3 individual face-to-face counselling sessions.  CG: usual care and were given American Cancer Society printed pamphlets and downloaded Web site information (cancer.org) related to PA after a cancer diagnosis. | Barriers self-efficacy (set of items);  Barrier interference (set of items);  Social support (set of items);   Outcome expectations (set of items);  Positive outcome expectations (set of items);  Fear of exercise (single item);  PA enjoyment exercise (single item). | PA 3 months of follow up (7-day accelerometer monitoring; GT1M Actigraph). | Only barrier interference and barriers self- efficacy demonstrated significant mediation effects. To quantify this effect, the improvements in barrier interference and barriers self-efficacy during the intervention mediated 39% and 19%, respectively, of the intervention effect on PA maintenance 3 months after intervention completion.  Social support, outcome expectations, positive outcome expectations, fear of exercise and enjoyment were not associated with PA. |
| Vallance et al., 2010  (Canada) | RCT | Breast cancer, I-IIIa; completed adjuvant therapy except hormone therapy; no current breast cancer. | 377 women; 58 years; 27.7± 5.6 kg/m2; N.R. | 4-armed randomized controlled trial with assessments at baseline, postintervention (12 weeks after randomization), and 6-month follow-up (6 months after postintervention  IG: All groups received a standard public health recommendation to perform 30 min of moderate-to-vigorous PA on 5 days of the week. Survivors meeting PA guidelines at baseline were encouraged to further increase their minutes and/or days spent engaged in PA. The Print materials (PM) group received the public health recommendation plus a copy of Exercise. for Health: An Exercise Guide for Breast Cancer Survivors.  The Pedometer group received the verbal public health recommendation plus a Digi- Walker SW-200 pedometer (ie, New Lifestyles Inc, Lee’s Summit, MO, USA) and a 3- month step calendar to record their daily steps during the intervention. The Combination group received both interventions (ie, Exercise for Health resource, pedometer, and step calendar) plus the verbal public health recommendation | HRQoL and fatigue (FACT-B);  TPB assessment (Intention; self-efficacy; Controllability; Attitudes (Instrumental and affective ); Injunctive Norm; Descriptive Norm; set of items). | PA at 6 months of follow-up (min/week MVPA; LTEQ). | Associations Between Patient-Rated Outcomes and 6-month Follow-up PA   Meeting guidelines was associated with increased Baseline HRQoL, baseline Fatigue, Postintervention HRQoL, postintervention fatigue.  Associations Between Postintervention Theory of Planned Behavior Constructs and 6-month Follow-up PA:  Intention, self-efficacy, Controllability, instrumental attitude, were positively associated with comply with guidelines.  Affective attitude, Injunctive norm, descriptive norm were not associated with comply with guidelines. |
| Winger et al. 2014  (USA) | RCT | Breast, Prostate, and Colorectal Cancer, five or more years postdiagnosis with no evidence of progressive disease or second primaries | 641; 54.4% women; 73.6 ± 5.1 years; N.R. | IG: A year-long program of telephone counselling and tailored mailed print diet and exercise materials based on social cognitive theory and the trans-theoretical model.  CG: waiting list | Physical function (ten-item physical function subscale of the MOS SF-36)  Basic and Advanced Lower Extremity Function (basic and advanced lower extremity subscales of the LLFDI)  Mental health (14-item mental health summary measure of the MOS SF-36) | Strength exercise (number of days and minutes per day of strength exercise over the past week)  Endurance exercise (number of days and minutes per day of endurance exercise over the past week.)  Post 1 year intervention. | Physical function and mental health were positively associated with strength exercise  Basic lower extremity function and advanced lower extremity function were not associated with strength exercise   Physical function, basic lower extremity function and advanced lower extremity function were associated with endurance exercise.  Mental health was no associated with endurance. |
| Frensham et al., 2018  (Australia) | Quasi-randomized  controlled trial | Multiple cancers, not be currently receiving active treatment such  as surgery, chemotherapy, or radiotherapy; | IG: 46; 54.3% women; 65.2 ± 9.3 years; N.R. CG: 45; 48.9% girls; 66.1 ± 9.4 years; N.R. | Participants used a pedometer to monitor the number of steps taken each day and recorded this information on the step log on the STRIDE website (12 weeks and 3-month follow-up)  CG: waiting list | Motivation (PAMA); Barriers self-efficacy (PAMA); Relapse self-efficacy (PAMA). | Steps (New-Lifestyles NL-1000 pedometer; New Lifestyles Inc, Lees Summit, Missouri) | Relapse self-efficacy and Barrier self-efficacy were positively associated with steps. No associations between motivations and steps |
| Morielli et al., 2018  (Canada) | prospective, single-arm intervention study | Rectal Cancer, receiving long-course  NACRT followed by definitive surgery | N=18; 33% women; 57.5 ± 10.4 years; 28.7 ± 4.2 kg/m2 | Supervised aerobic exercise program began at the initiation of NACRT and continued during NACRT. The unsupervised aerobic exercise program began after NACRT until 1–2 weeks before surgery. During NACRT, the primary goal of the exercise intervention was to attend three sessions per week for the 6 weeks of NACRT. After NACRT, patients were asked to complete 150 min of moderate-intensity aerobic exercise per week. | Quality of Life (MOS SF-36)  Fatigue (FACT-F)  Stress (PSS)  Retrospective motivational evaluation of the supervised exercise program in which they were asked to look back and report how beneficial, enjoyable, supported, motivated, and difficult it actually was to complete the aerobic exercise intervention during their chemoradiotherapy. | Exercise adherence (Minutes of aerobic exercise per week; GLTEQ).  6 weeks. | No significant associations between adherence to supervised aerobic exercise and physical health, mental health, fatigue, stress, beneficial, enjoyable, support, motivation and difficulty.  Adherence to the unsupervised exercise was significantly better in patients who reported worse mental health. |
| Ungar et al., 2016  (Germany) | Experimental Randomized with no control | Multiple cancers, receiving out-  Patient therapy (acute or maintenance therapy) or finished his  Therapy not longer than 6 months ago | 67; 52% women; 55.45 ± 12.62 years; N.R. | IG 1: exercise intervention included counselling based on the Health Action Process Approach IG 2. stress management | PA Enjoyment (set of items);  Maintenance Self-Efficacy (set of items). | PA level (SQUASH)  0, 4 weeks after the intervention, 10 weeks after the intervention) | Baseline:  PA enjoyment and self-efficacy was not associated with PA  4 weeks after: PA enjoyment and self-efficacy were positively associated with PA  10 weeks after: Self-efficacy was positively associated with PA  PA enjoyment was not associated with PA |
| Basen-Engquist et al., 2013  (USA) | 6-month longitudinal study with intervention | Endometrial Cancer, at least 6 months post treatment with no evidence of disease Stage I, II, or IIIa | 100 women; 57.0 ± 11.01 years; 34.2 ± 9.4 kg/m2 | Each participant received an exercise recommendation tailored to her fitness level based on ACSM guidelines, provided by a masters-level exercise physiologist. The ultimate goal was for the survivor to work up to moderate-intensity exercise for at least 30 min a day on 5 or more days per week. Provided telephone counseling, print materials, and a pedometer | Morning assessments: EMA - Single item self-efficacy;  Positive and negative outcome expectations;  Exercise self-efficacy (no name);  Barriers self-efficacy (no name);  Positive and negative outcome expectations (no name). | PA (min/week; CHAMPS)  6 months | Exercise self-efficacy was positively associated with PA  Positive outcome expectations, barriers self-efficacy and Negative outcome expectations were not associated with PA |
| André et al., 2018 (study 2)  (France) | NRCT | Breast cancer, N.R. | 45 women; 68.6 ± 6.2 years; N.R. | Therapeutic patient education (TPE): helping patients acquire or maintain the competences they need to manage their life with a chronic disease as well as possible | Decisional balance (set of items);  Cancer-related barriers (experienced fatigue, depression, and lack of information - set of items); | Level of engagement in PA (NASA/JSC PA Scale that discriminates categories of PA levels: From 0 to 2, participants are considered as inactive; from 3 to 5, participants are considered active but below recommendations; from 6 to 7, participants are considered fully active. | Only changes in cancer-related barriers were predictors of changes in engagement. Pros and cons were not predictors. |
| Stone et al., 2019  (Canada) | Cohort | Prostate cancer, from pre-diagnosis to 2 years post-diagnosis stage II–  IV | N = 817 men; N.R.; N.R. | NA | Quality of Life (MOS SF-36 component summary scores for physical and mental component);  Friend support (yes or no);  Familly support (yes or no) | PA (LTPAQ) | Friend support was a predictor of short- and long-term physical activity Family support was no associated.  Less than average scores on the physical quality of life component were found to be significantly associated with being a non-exerciser compared to being maintainer or adopter, while less than average scores on the mental quality of life component was only associated with being a non-exerciser compared to adopter |
| Wilson et al., 2006  (USA) | Cohort | Multiple cancers; N.R. | 220; 53% women; Men: 64.83 ± 14.98 years, Women: 59.80 ± 16.52 years; Men: 26.92 ± 6.01 kg/m2, Women: 28.78 ± 7.07 kg/m2 | NA | Autonomous and controlled motives (TSRQ-PQ);  Outcome expectation (set of items) | PA (items were adapted from the BRFSS to measure the frequency (per week) and duration (minutes per session) of time spent engaging in MVPA) | Autonomous motives were a positive predictor of MVPA and controlled motives a negative predictor after controlling for demographic variables.  Outcome expectations was not associated with MVPA. |
| Culos-reed et al., 2005  (Canada) | Prospective, observational | Breast Cancer, N.R. | 56 women; 52.89 ± 7.61 years; N.R. | NA | Attitude, subjective norm, PBC (set of items) | PA levels early season (approximately 3–4 weeks after the beginning of regular training) and at late season (approximately 2 weeks prior to end of regular training) (LSI-GLTEQ) | Early season (n=109):  Perceived behaviour control was positively associated with PA.  Subjective norm, attitudes were not associated with PA   Late season (n=56)  PBC was positively associated with PA  Subjective norm, attitudes were not associated with PA   Between early and late season  Attitudes was positively associated with PA  Subjective norm and PBC were not associated with PA |
| Karvinen et al., 2009  (Canada) | Prospective | Bladder Cancer, any stage | 397; 25.3% women; 70.2 ± 11.2 years; N.R. | NA | Exercise intention, attitudes (instrumental and affective), subjective norm, perceived behavioural control (set of items) | Exercise behaviour over the past 3 months at the 3-month follow-up (LSI-GLTEQ. | PBC, intention, affective and instrumental attitude was positively associated with exercise behaviour  Subjective norm were not associated with exercise behaviour. |
| Phillips and McAuley., 2013  (USA) | Prospective, Longitudinal | Breast cancer, N.R. | Total Sample: 1527 women; 56.2 ± 9.4 years; N.R. | NA | Self-efficacy (EXSE);  Outcome Expectation (MOEES);  Fatigue (FSI)  Social support (SSE). | PA (min/week; GLTEQ); | At baseline, women with higher social outcome expectations participated in more PA. Social support and fatigue were not associated with PA. At 6-month follow-up, breast cancer survivors whose self-efficacy increased had significant increases in PA; Social support, fatigue and outcome expectation were not associated with PA. |
| André et al., 2018 (study 1)  (France) | Cross-sectional | Breast cancer, N.R. | 139 woman, <50 years = 15 %, 50-64 years = 43.9%; ≥ 65 years = 40.3% | NA | Decisional balance (set of items)  Cancer-related barriers (experienced fatigue, depression, and lack of information - set of items); | Level of engagement in PA (NASA/JSC PA Scale that discriminates categories of PA levels: From 0 to 2, participants are considered as inactive; from 3 to 5, participants are considered active but below recommendations; from 6 to 7, participants are considered fully active. | Pros and cons were identified as significant predictors of PA engagement level. Adding barriers to the model brought up the explained variance. Pros and cons remained significant. |
| Bélanger et al., 2013  (Canada) | Cross-sectional | Multiple cancers, N.R. | 588; 70% women; 38.2 **±** 5.6 years; 26.5 **±** 5.7 kg/m2 | NA | Quality of Life (MOS SF-36)  Perceived Stress (PSS)  Self-esteem (RSES)  Depression (CES-D) | Sports participation (set of items; dichotomic variable, yes or no). | Sports participation was positively associated with lower depression, higher self-esteem, and lower perceived stress. Sports participation was also associated with higher scores on the physical and mental components of quality of life. |
| Coups et al., 2009  (USA) | Cross-sectional | Lung Cancer, stage IA or IB; underwent surgical resection in the previous 6 years; no current evidence of any cancer | 175; 63% women, 68.7 ± 9.6 years; N.R. | NA | PA self-efficacy (BSES)  PA outcome expectations (DBQ);  Perceived barriers to PA (set of items);  Perceived social support (SES). | Total weekly minutes of MVPA (modified GLTEQ);  Past month engagement in leisure walking (Leisure walking index from the YPAS). | PA self-efficacy and outcome expectations were positively associated with MVPA.   PA self-efficacy was positively but marginally associated with leisure walking.   PA outcome expectations were also positively associated with leisure walking.  No associations between PA outcomes and perceived social support, or perceived barriers to PA. |
| Blanchard et al., 2002  (Canada) | Cross-sectional | Breast and prostate cancer, N.R. | Breast: 83 women; 61.75 ± 12.2 years; N.R.; Prostate: 46 men; 68.13 ± 7.07 years; N.R. | NA | Attitude (set of items);  Perceived behavioural control (set of items);  Subjective norm (set of items);  Intention (set of items). | PA (LSI-GLTEQ) | Intention, attitudes, subjective norm and perceived control were positively associated with PA for both populations. |
| Collins et al., 2018  (Canada, USA, UK, Ireland, Australia,  and New Zealand) | Cross-sectional | Multiple cancers, N.R. | 147; 81.6% women; 31.9 ± 5.7 years; N.R. | NA | Quality of life (FACT-G: physical wellbeing,  social well-being, emotional well-being, and functioning  well-being; and MOS SF-36: Vitality, physical functioning, bodily pain, general health perceptions, physical role functioning, emotional role functioning, social role functioning, and mental health). | PA by intensity (LSI-GLTEQ) | Light PA was not associated with physical wellbeing, social well-being; emotional well-being, Functional wellbeing and QoL score.  Moderate PA was not associated with physical wellbeing, social well-being; emotional well-being, Functional wellbeing and QoL score.  Vigorous PA was positively associated with physical wellbeing, but not with social well-being; emotional well-being, Functional wellbeing and QoL score.  Resistance training was positively associated with functional well-being, but not with physical wellbeing, social well-being; emotional well-being, and QoL score.  MVPA was positively associated with physical wellbeing, but not with Social well-being; emotional well-being, Functional wellbeing and QoL score  SF36 Light PA were not associated with physical functioning, role limitations due to physical problems, bodily pain, general health, vitality, social role functioning, role limitations due to emotional problems, mental health, physical component score, mental component score.  Moderate PA were associated with physical functioning and physical component score, but not with role limitations due to physical problems, bodily pain; general health perceptions, vitality; social role functioning, role limitations due to emotional problems, mental health, mental component score.  Vigorous PA was positively associated with physical functioning, bodily pain, general health perceptions, vitality, and physical component score, but not with role limitations due to physical problems, social role functioning, role limitations due to emotional problems, mental health and mental component score  Resistance training was positively associated with general health perceptions, but not with physical functioning, role limitations due to physical problems, bodily pain, vitality; social role functioning, role limitations due to emotional problems, mental health, physical component score, mental component score.  MVPA was positively associated with physical functioning, role limitations due to physical problems, bodily, general health perceptions, vitality and physical component score, but not with social role functioning, role limitations due to emotional problems, mental health and mental component score. |
| Farrokhzadi et al. 2016  (Australia) | Cross-sectional | Gynecological Cancer (ovarian, emdometrial, cervical, other); N.R. | 101 women; 58 years; 27 kg/m2. | NA | Barriers to PA (set of items) | Post-diagnosis leisure-time PA (min/week; AAQ) Usual pre-diagnosis PA (GLTEQ) | There were negative associations between PA, and lack of interest, “Too tired”, and “Not well enough”.  No associations with family encouragement; doctor encouragement and fear of injury. |
| Finnegan et al., 2007  (USA) | Cross-sectional | Various types of childhood cancers, at least two years beyond completion  of cancer therapy | 117; 68% women; 24 ± 5 years; N.R. | NA | Autonomous motivation scale (BREQ-2);  PA self-efficacy (set of items);  Decisional balance (DBSPA);  Self-reported worries scale (affective response) (set of items) | PA stages of change were measured with a single-item ((a) precontemplation (not active and not planning to start in the next six months), (b) contemplation (not active but planning to start in the next six months), (c) preparation (not active but planning to start in the next 30 days), (d) action (active for less than six months), or (e) maintenance (active for more than six months) | Survivors who had higher scores for autonomous motivation were more likely to be active than survivors with lower autonomous motivation scores. Similarly, survivors with higher self-efficacy scores were more likely to be active than survivors with lower self-efficacy scores. Higher scores on the PA cons measure were associated with a decreased likelihood of being physically active. |
| Frikkel et al., 2020  (Germany) | Cross-sectional | Multiple cancers, Stage IV, Outpatients with metastatic cancer. | 141; 60% women; 60 ± 11 years; N.R. | NA | Motivation to PA (set of item);  Fatigue (FACT-F);  Depression (PHQ- depression scale);  Interest in exercise, motivation to PA, fear of adverse events of PA, clinical depression, knowledge of positive impact of PA (set of items) | PA (dichotomic variable: being physically active at least once a week vs physically inactive [i.e., no activity at all]. | The active group showed significantly higher motivation to PA and interest in an exercise program, then the inactive group.  Fatigue, depression, fear of adverse events of PA. knowledge were not associated to PA. |
| Kampshoff et al., 2016  (Netherlands and Australia) | Cross-sectional | Brest Cancer, completed primary cancer treatment | 484 women; 54.5 ± 9.2 years; 27.0 ± 5.2 kg/m2 | NA | Self-efficacy (set of items)  Social support (set of items)   Outcome expectations (set of items) | Accelerometer (ActiTrainer; Actigraph) Pedometer (Yamax Digi-Walker, SW200) | Self-efficacy and social support and outcome expectations were positively associated with PA. |
| Kang et al., 2014  (South Korea) | Cross-sectional | Colorectal cancer, stage I-III | 427; 37% women; 46.6% were over 60 years old and 47.1 % under 60 years | NA | Barriers (EBQOA) | PA (min/week; EQoLQ) | Participating in PA was negatively associated with fatigue, poor health, lack of interest, lack of motivation, stress from doing exercise, fear of what others think of me doing exercise.  No associations with fear of injury, fear of adverse effect on cancer site or treatment, family opposition.  Meeting guidelines are negatively associated with fatigue, poor health, lack of interest, fear of injury, fear of adverse effect on cancer site or treatment, stress from doing exercise.  No associations with lack of motivation, fear of what others think of me doing exercise, family opposition. |
| Karvinen et al., 2007  (Canada) | Cross-sectional | Endometrial cancer, within the last 15 years. | 354 women; 64.5 ± 10.6 years; 29.3  ± 6.6 kg/m2 | NA | Intention, attitudes (affective and instrumental), subjective norm, perceived behavioural control, self-efficacy (Set of items) | PA (min/week; modified LSI-GLTEQ) | Intention, instrumental attitude, affective attitude, subjective norm, self-efficacy, and perceived control were positively associated with PA.  Results of the multiple regression procedures indicated that TPB explained 34.1% of the variance in exercise behaviour with intention and self-efficacy providing independent associations. |
| Keogh et al., 2010  (New Zealand) | Cross-sectional | Prostate cancer on androgen-deprivation therapy (ADT) | 84 men; 78.4 ± 8.21 years; N.R. | NA | 47-item inventory probing intention to be physically active, perceived control of factors that prevent or encourage physical activity, attitudes towards PA, and pressures from significant others to be physically active or not | PA (min/week; RAPA) | Perceived control was positively associated with PA.  Attitude, subjective norms were not associated with PA. |
| Krok-Schoen et al., 2021  (USA) | Cross-sectional | Older female (>65y) cancer survivors, all cancer types | 171; 73.5 ± 8.4 years; 26.54 ± 6.22 kg/m2 | NA | HRQoL (RAND-36: the 36-item health survey) - physical health (composite score PCS) and mental health (composite score MCS) | PA (Personal Habits Questionnaire - mild, moderate or strenuous exercise habits) | Physical HRQoL was positively correlated with engaging in strenuous (r = 0.31, p < 0.001) and moderate  physical activity (r = 0.35, p < 0.001).  Controlling for demographic and  clinical factors, engagement in moderate physical activity was  associated with higher physical HRQoL (β = 0.42, p = 0.004;  Model: F = 9.51, p = 0.004).  No correlation for mental HRQoL and moderate or strenuous exercise.  No correlation for physical or mental HRQoL and mild exercise. |
| Krok-Schoen et al., 2022  (USA) | Cross-sectional | Breast cancer survivors | 3710; 78.8 ± 5.9y | NA | Symptom score (LILAC one year follow-up survey (Form 370))  Pain (LILAC one year follow-up survey (Form 370))  Self-rated health (LILAC one year follow-up survey (Form 370))  Global QoL (LILAC one year follow-up survey (Form 370))  Physical functioning (LILAC one year follow-up survey (Form 370))  Social support (single item from MOS-Social Support Questionnaire) | Self-reported physical activity (own study questionnaire - LILAC one year follow-up survey (Form 370)) | Higher reported social support, had lower symptom burden, higher self-rated health, higher quality of life, higher physical functioning, no pain and no depressive symptoms had significantly higher duration of physical activity (all p < 0.05) in bivariate analysis.  Social support, self-rated health and physical functioning were positively correlated with physical activity  duration in multivariate analysis.  Symptom burden, quality of life and depressive symptoms were not associated with physical activity  duration in multivariate analysis. |
| Kucukvardar et al., 2021  (Turkey) | Cross-sectional | Colorectal cancer on stage II–III and have completed cancer treatments, including  surgery, radiation therapy, and chemotherapy, and stopped all  medical treatments at least 3 months before the beginning of the study | 47; 31.9%; 58.19 ± 12.03 years; 27.36 kg/m2 | NA | Fatigue (Brief Fatigue  Inventory)  QoL (36-item Short Form  Health Survey: physical  functioning, role limitations due to physical problems,  bodily pain, general health perceptions, vitality, social functioning,  role limitations due to emotional problems, and mental health) | MVPA (METs/min/week; IPAQ) | Severity of fatigue, the impact of fatigue on daily functioning, role  limitations caused by physical health problems, physical functioning, general health perceptions and vitality were positively associated with MVPA.  Bodily pain, social functioning, role emotional, and mental health were not associated with MVPA.  In multiple linear regression general quality of life and severity of fatigue were positively associated with MVPA. |
| Lee et al., 2018  (South Korea) | Cross-sectional | Colorectal Cancer, stage-I-IV, currently using or had completed therapy | 251; 31% women; 62.7 ± 10.9 years; N.R. | NA | Self-leadership (ASLQ; behaviour awareness and volition, task motivation, constructive cognition);  Perceived Social Support (set of questions: family’s participation and involvement, family’s rewards, family’s criticism and friend’s exercising together. | 7-day exercise diaries in minutes per week of at least moderate aerobic exercise that consumed at least 4 metabolic equivalents (3.5 mL O 2/ kg/min). Maintaining exercise was measured in persistence of the exercise for 6 months. | The use of rewards by family for performing exercise was associated with performing more exercise and maintaining exercise for more than 6 months. Self-leadership and the other subscales from perceived social support were not associated. |
| Lee et al., 2020  (South Korea) | Cross-sectional | Colorectal Cancer, stage-I-IV, within the 2 previous years. Currently using or had completed therapy | 251; 31% women; 62.7 ± 10.9; N.R. | NA | Self-leadership (ASLQ; behaviour awareness and volition, task motivation, constructive cognition); | 7-day exercise diaries in minutes per week of at least moderate aerobic exercise that consumed at least 4 metabolic equivalents (3.5 mL O 2/ kg/min). Maintaining exercise was measured in persistence of the exercise for 6 months. | Performing moderate-intensity aerobic exercise more than 150 min/week correlated positively with higher self-leadership in the following subscales: behaviour awareness and volition, task, and constructive cognition.  Maintenance of moderate-intensity aerobic exercise for 6 months had positive correlations with higher self-leadership in the following subscales: behaviour awareness and volition, task motivation, and constructive cognition. |
| Lesser et al., 2021  (Canada) | Cross-sectional | Multiple cancer, with diagnosis. | 114; 76.3%; 53.6 ± 14.06 years; N.R.. | NA | Anxiety (Generalized Anxiety Disorder (GAD-7) scale);  Fatigue (FACIT-fatigue  scale);  Happiness (subjective happiness scale)  Barriers and facilitators (no named) | MVPA (Godin Leisure Questionnaire; min/wk) | Motivation, PA benefits, enjoyment , confidence with PA.  Anxiety, fatigue, happiness, difficulty and social support were not associated with MVPA. |
| Murray et al., 2019  (USA) | Cross-sectional | Multiple cancers, at least one-year post-primary  treatment for cancer | 121; 88% women; 57.02 ± 12.29; N.R. | NA | Cognitive beliefs (set of questions);  Subjective norms (set of questions);  Perceived behavioural control (set of questions);  Benefits and barriers (set of questions);  Affective predictors: affective associations (set of questions);  Worry (set of questions);  Implicit affect (AMP Procedure Jarvis, 2012). | Total PA over a one-week period (BPAT and GLTEQ)   Assessed by telephone call (2 weeks to 6 months post session) | Call-back PA: Positive Affective, Cognitive Beliefs, Subjective norms, Perceived Control, Perceived benefits were positively associated with PA. Negative affective, worry about PA and perceived barriers were negatively associated with PA. In regression analysis, two cognitive variables served as independent predictors: cognitive beliefs and perceived barriers. Two affective variables were independent predictors: positive affective associations, and worry. When the affect variables were entered into the model on Step 2, none of the cognitive variables continued to predict behavioural estimates |
| Ng at al., 2021  (USA) | Cross-sectional | Multiple cancers, survivors | 200 (110 women, 55%); >18y | NA | Exercise Barriers (adapted Survey of Exercise Barrier Scale)  Physical Function (Patient-Reported Outcome Measurement Information System–Short Form (PROMIS-SF) v1.0–Physical Function 12a)  Symptom burden / well-being (Edmonton Symptom Assessment System (ESAS-FS)) | PA (Stanford Patient Education Research Center Exercise Behaviours Survey) | Higher ESAS well-being score (ie, worse well-being) was associated with a lower chance of reporting >60 minutes of exercise (OR 0.731; P = 0.006)  Among the 68 participants who reported >150 minutes of  exercise, a survey response of ‘not often’ was significantly more common for lack of interest (n=62, 91%; P=0.003), lack of self-discipline (n=58, 85%;P=0.001), lack of enjoyment (n=58, 85%; P=0.054). They also more frequently reported ‘not often’ to experiencing symptoms of fatigue (n=47, 69%; P=0.005) and pain and discomfort (n=48, 71%; P=0.007).  Those who reported >150 minutes of exercise reported fewer barriers (P=0.0001). |
| Ott et al., 2004  (USA) | Cross-sectional | Breast cancer, completion of treatment (except Tamoxifen), 6 months previous for Stage I or II | 23 women; 54.8 ± 7.2 years; 27 kg/m2 | NA | Self-efficacy (set of items) Change Process (set of items) Decisional Balance (set of items) | Adherence to exercise (0, 2m, 6m): A two-part adherence journal was used by participants to document exercise frequency, repetitions, sets, and amount of weights used for ankles and arms on a weekly basis. | Self-efficacy, benefits (pro) and costs (cons) had no significant relationship with exercise adherence at 6 months.  There were no significant relationships between behavioural or cognitive change processes and adherence to exercise. |
| Park et al., 2020  (South Korea) | Cross-sectional | Colorectal and breast cancer, Stage 0-IV completed primary and adjuvant treatments | 224; 80% women; 54.4 ± 7.8 years (men), 51.5 ± 8.2 (women); N.R. | NA | QoL (EORTC QLQ C-30) | Light PA, Moderate PA, and Vigorous PA (LSI-GLTEQ) | MVPA was positively correlated with general quality of life, physical functioning, emotional functioning, cognitive functioning, fatigue, pain. No associations with role functioning and social functioning. Cognitive functioning was no longer associated after adjustment for confounding factors.  Total PA was positively correlated with general QoL, physical functioning, role functioning, emotional functioning pain and fatigue. No associations with cognitive functioning and social functioning. After adjustment, it remained correlated with general QoL, physical functioning, fatigue.  No associations between mild PA and all variables. After adjustment, it was positively correlated with role functioning. |
| Paxton et al., 2019  (USA) | Cross-sectional | Breast Cancer | 267 women; 54 years; 30.4 ± 5.9 kg/m2 | NA | Positive and negative outcome expectations, motivational self-efficacy, pre-actional Self-efficacy, coping self-efficacy, and relapse prevention Self-efficacy (set of items)  PA barriers (Set of questions) Perceived functional status and disability (modified LLFDI) | Recreational walking and light, MPA, VPA, and guidelines compliance (WHI-PHQ). | The four variables that were retained as the most important correlates of PA compliance were, advanced lower function, and barriers to PA. Compliance with PA guidelines was higher when had a mean barriers to PA score of ≤ 28.  Basic lower extremity function, upper extremity function, fatigue, physical role disability, social role disability, intention, positive and negative outcome expectations, motivational Self-efficacy, pre-actional Self-efficacy, coping self-efficacy, and relapse prevention self-efficacy were not associated with PA. |
| Peddle et al., 2008a  (Canada) | Cross-sectional | Colorectal Cancer, Completed adjuvant therapy for at least one year, had no evidence of recurrent disease | 413; 46% women; 60.7 ± 7.4 years; N.R. | NA | Quality of life (FACT-C; subscales: physical well-being, functional well-being, emotional well-being, social well-being, disease-specific subscale)  Fatigue (FACT-F)  Trial outcome index (colorectal), a composite measure of physical well-being, functional well-being, and disease-specific subscale trial outcome index-fatigue, a combined score of physical well-being, functional well-being, and fatigue) | Exercise behaviour (modified LSI-GLTEQ).  Participants were categorized as meeting or not meeting public health exercise guidelines recommended by the American College of Sports Medicine and the American Cancer Society. | Those meeting public health exercise guidelines reported better quality of life, fatigue, trial outcome index, physical well-being, and functional well-being, than those not meeting guidelines.  No significant results for emotional well-being, social well-being, and additional concerns. |
| Peddle et al., 2008b  (Canada) | Cross-sectional | colorectal cancer, completed adjuvant therapy for at least 1 year, had no evidence of recurrent disease | 413; 66% women; 60 ± 7.5; 29.0 ± 5.5 kg/m2 | NA | Motivational (BREQ-2);  Perceived autonomy support (PAS);  Psychological need satisfaction in exercise (PNSE; competence, relatedness, autonomy support ; | Exercise behaviour (min/week; modified LSI-GLTEQ) | Amotivation was negatively associated with exercise behaviour. Introjected Regulation, identified regulation and Intrinsic regulation were positively associated with exercise behaviour  External regulation was not associated with exercise behaviour.  PNSE competence, PNSE relatedness, Perceived autonomy support, PNSE autonomy were positively associated with exercise behaviour |
| Price et al., 2021  (Canada) | Cross-sectional | Multiple cancers, cancer, completed primary treatment for cancer | 123; 82.9%; 50.1 ± 15.5; 27.55 ± 6.31 | NA | Health beliefs (no named; perceived susceptibility to cancer recurrence and health problems, perceived severity of cancer recurrence and health problems, perceived benefits of PA for the prevention of cancer recurrence and health problems, perceived barriers to engaging in PA, and PA barrier self-efficacy ) | MVPA (Min/week; modified GLTEQ) | Perceived benefits of PA, PA barrier self-efficacy and less perceived barriers to PA were significantly associated with MVPA. Perceived susceptibility, perceived severity were not significant.  Perceived barriers to PA were not associated with MVPA in multivariate analysis. |
| Ribeiro et al., 2018  (Brazil) | Cross-sectional | Breast Cancer | N=102 women; 58.2 ± 10.3 years; 29.1 ± 4.8 kg/m2 | NA | Body Dissatisfaction (Silhouete scale for adults) | PA Level (Baecke questionnaire): general score determining the total PA level. | No associations with body dissatisfaction were observed. |
| Robertson et al., 2018  (USA) | Cross-sectional | Multiple cancers, N.R. | 1006; 59% women; 18-75+ years | NA | Motivations for PA: Extrinsic, Intrinsic (Set of items) | Typical weekly minutes of aerobic MVPA by multiplying (1) weekly frequency of MVPA with (2) typical time spent in MVPA. | Generalized linear regression models indicated that those who were more motivated by “getting enjoyment from exercise” reported more MVPA than those who were less motivated by this factor. Conversely, those who were more motivated by “concern over the way you look” reported less MVPA than those who were less motivated by this factor. |
| Rogers et al., 2008 (Support Care Cancer)  (USA) | Cross-sectional | Head and Neck Cancer, stage I, II, III, IV with recurrent disease, or off treatment at time of the survey | 59; 17% women; 58 ± 12.8 years; 25.9 ± 6.5 kg/m2 | NA | Barrier Self-efficacy, Task self-efficacy, social support, perceived PA barriers, PA enjoyment (set of items)  Depression (CES-D) | PA by intensity (modified GLTEQ)  Total exercise minutes from all intensities were calculated for prediagnosis and current levels. | The strongest correlates of PA included, task self-efficacy, perceived barriers, PA enjoyment. Stepwise regression demonstrated that enjoyment had independent associations with PA.  Barrier self-efficacy, social support, depression and role model were not significantly associated. |
| Rogers et al., 2015  (USA) | Cross-sectional | Head and Neck Cancer, N.R. | 101; 27% women; 60 ± 12 years; N.R. | NA | Barriers self-efficacy (BSES= barriers self-efficacy scale);  Barriers (PBIS);  Outcome expectations (OES);  Exercise enjoyment (set of items) | PA adherence (GLTEQ; MVPA was used to determine if current exercise recommendations for ≥150 weekly minutes were met;) | Meeting the guidelines was associated with barriers for self-efficacy in all subscales, motivational subscale and health subscale, with outcome expectations, with exercise enjoyment.  Also, meeting guidelines were negatively associated with barriers interference in all subscales, motivational subscale, physical health subscale |
| Short et al., 2014  (Australia) | Cross-sectional | Breast cancer, post-treatment | 330 women; 55.86 ± 7.86 years; BMI of over 25 (60.6 %). | NA | HRQoL (FACT-B);  Fatigue (FACIT-F);  Outcome expectations (set of items);  Outcome expectancies (set of items);  Task self-efficacy for resistance training activities (set of items)  Barrier self-efficacy (set of items)  Behavioural capability (set of items)  Social support (SSEHS) | Resistance training past month (was assessed using a  previously developed item designed to determine whether  or not respondents were meeting the public health guidelines  for resistance training (defined as resistance training  performed on 2 or more days a week that includes exercises  involving all major muscle groups). | Meeting guidelines were positively associated with outcome expectations, outcome expectancies, task self-efficacy, barrier self-efficacy, behavioural capability, family social support, friend social support.  Fatigue and quality of life were not associated. |
| Skiba et al., 2022 | Cross-sectional | Self-reported history of cancer. | Overall 1864; 56% women; 65.05 ± 13.65 years; BMI over 25 (67.3%) | NA | Self-efficacy (manage health)  Risk belief (cancer) | PA was assessed using the following questions: ‘‘In a typical week, how many days do you do any physical activity or exercise of at least moderate intensity, such as brisk walking, bicycling at a regular pace, and swimming at a regular pace  (do not include weightlifting)?’’ and ‘‘On the days that you do any physical activity or exercise of at least moderate intensity, how long do you typically do these activities?’’ (aerobic PA) and ‘‘In a typical week, outside of your job or work around the house, how many days do you do leisure-time physical activities specifically designed to strengthen your muscles such as lifting weights or circuit training (do not include cardio exercise such as walking, biking, or swimming)?’’ (resistance PA). Continuous responses were dichotomized to meeting recommendations (>150 weekly minutes for aerobic PA and > days weekly for resistance PA) according to ACS guidelines. Aerobic and resistance PA were analyzed separately. | Select health beliefs were associated with adherence to recommendations for FV and resistance and aerobic PA.  Self-regulation was consistently associated with meeting recommendations. High self-efficacy was associated with meeting aerobic PA recommendations. Self-efficacy and self-regulation were positively associated with Modified ACS Adherence score. |
| Smith et al., 2018  (USA) | Cross-sectional | breast cancer, at least one year post-treatment. | 193 woman; 55.6 ± 12.8 years; N.R. | NA | HRQoL (PROMIS 2016; one dimension of mental health (depression) and four dimensions of physical health (fatigue, pain interference, pain intensity, and physical functioning)) | PA was based on meeting the recommendation of >150 min total PA/wk as sufficient and >0 minutes but <150 minutes per week as insufficient (BRFSS) | Meet guidelines were positively associated with physical functioning and pain intensity. Depression, fatigue, and pain interference were not associated |
| Smith-Turchyn et al., 2021 | Cross-sectional | confirmed diagnosis of cancer (any type and stage) | 72 participants; 65 ± 10.3 years | NA | Perceived Exercise Benefit:  Cancer-Related  Fatigue  Interest in Exercise  Exercise Motivators  Exercise Limitations  Exercise Facilitating Factors  PA Goals | Moderate-to-Vigorous Intensity PA  (Minutes/Week)  Meeting AET Guidelines  Meeting RET Guidelines | Those reporting fewer exercise limitations reported meeting aerobic exercise guidelines in greater  proportion than those reporting more limitations (p = 0.04), as did those taking fewer medications (p = 0.01), those with localized disease (p = 0.02), and those with lower levels  of fatigue (p = 0.02). |
| Stevinson et al., 2009  (Canada) | Cross- sectional | Ovarian Cancer, diagnosis of histologically confirmed ovarian cancer | 112 women, 60.2 ± 12.6 years; 27.1 ± 5.4 kg/m2 | NA | Intention, Attitude (instrumental and affective), PBC, Subjective norm (set of items) | Meeting vs not meeting the guidelines; (1) completely sedentary (no moderate or strenuous PA), (2) insufficiently active (<150 minutes of moderate/strenuous activity and <60 minutes of strenuous activity), (3) within guidelines (150 to 300 minutes of moderate/strenuous or 60 to 120 minutes of strenuous activity), and (4) above guidelines (≥300 minutes of moderate/strenuous or ≥120 minutes of strenuous activity).  (LSI-GLTEQ) | There is a positive association between PA and intention, instrumental attitude, affective attitude, injunctive norm, descriptive norm, Perceived behaviour control.   The hierarchical multiple-regression analysis, indicating that 36% of the variance in PA behaviour was explained with intention being the sole independent correlate |
| Tabaczynski et al., 2019  (Canada) | Cross-sectional | Kidney Cancer | 651; 38% women; 64.4 ± 11.0; 28.6 ± 5.2 kg/m2 | NA | Theory of Planned Behaviour (set of items; affective attitude; instrumental attitude; injunctive norm; descriptive norm; perceived behaviour control, intention) | % Meeting aerobic-only (i.e., ≥150 min/week of MVPA), Strength training-only (i.e., ≥ 2 days/week of any duration), and combined guidelines (i.e., ≥ 150 min/week of MVPA and ≥ 2 days/week of Strength training) was calculated based on the ACSM guidelines (Modified LSI-GLTEQ) | (a) meeting the aerobic-only guideline was positively associated with higher intentions; no associations with affective attitude, instrumental attitude, injunctive norm, descriptive norm, perceived behavioural control (b) meeting the Strength training -only guideline was positively associated with higher intentions, lower PBC; No associations with affective attitude, instrumental attitude, injunctive norm, descriptive norm, (c) meeting the combined guidelines was positively associated with higher intention, higher instrumental attitudes. No association with affective attitude, injunctive norm, descriptive norm and Perceived behavioural control |
| Wurz and Brunet, 2019  (Canada) | Cross-sectional | Multiple cancers, had completed cancer treatment within the past 5 years | 87; 75% women; 32.90 ± 4.38 years; 25.41 ± 6.08 kg/m2 | NA | Physical self-perceptions and physical self-esteem (PSDQ‐S; strength, endurance, appearance, body fat);  General self-esteem (RSE);  Self-efficacy for PA (modified single‐item version of the ESE); | PA (min/week; modified LTEQ) | Self-efficacy was positively associated with PA. Physical self-perception and physical self-esteem were not associated |
| Yan et al., 2021  (China) | Cross-sectional | Cancer survivors, any type | 1546; 73.16% women | NA | HRQoL (EORTC QLQ-C30) | High PA level (HPAL)  ((a) 7 or more days of any combination of walking, moderate- or vigorous-intensity PA accumulating≥3000 MET-min/week, or (b)≥3 days of vigorous-intensity PA and accumulating≥1500 MET-  min/week)  Moderate PA level (MPA)  ((a)≥3 days of vigorous-intensity PA with≥20 min per day, or (b)≥5 days of moderate-intensity PA and/or walk≥30 min per day, or (c)≥5 days of any combination of walking, moderate or vigorous-intensity PA accumulating a total of≥600  MET-min/week)  Low PA level (LPAL)  (no activity or those didn’t meet the criteria for MPAL or HPAL)  (IPAQ) | CSs with HPAL reported higher  score of physical function, role function, and emotional  function than those with LPAL.  No association for cognitive function, social function, global health/HRQoL and fatigue. |

SD= Standard deviation; BMI= Body mass index; PA= Physical activity; RCT= Randomized Control Trial; IG= Intervention group; CG= Control group; PASA-NIH= Physical Activity State Assessment measure developed for the NIH Behavior Change Consortium; SCFS= Schwartz Cancer Fatigue Scale; MOS SF-36= Medical Outcomes Study Short-Form 36; CHAMPS= Community Healthy Activities Model Program for Seniors Physical Activity Questionnaire for older adults; N.R.= Not reported; FACT-An= Functional Assessment of Cancer Therapy-Anemia; TOI-An= Trial Outcome Index-Anemia; CES-D = Center for Epidemiological Studies Depression Scale; SSAI=Spielberger State Anxiety Inventory; QoL= Quality of life; TPB= Theory of Planned Behaviour; PBC= Perceived behavioral control; NEO 4FI= NEO Five Factor Inventory; LSI= leisure score index; GLTEQ= Godin Leisure-Time Exercise Questionnaire; HRQoL= Health related quality of life; MDI= Multidimensional Fatigue Inventory; EORTC-QOL-C30= European Organization for Research and Treatment of Cancer Quality of Life Questionaire; SQUASH= Short Questionnaire to Assess Health-enhancing physical activity; MVPA= Moderate to vigorous physical activity; SSQ= Social Support Questionnaire; SESWL= Self-Efficacy Scale for Weight Loss; MAQ= Modified Activity Questionnaire; MET= Metabolic equivalent of task; CSEBSES= Cancer survivors’ exercise barriers self-efficacy scale); SSES= The Social Support for Exercise Survey; IPAQ= The International Physical Activity Questionnaire TSRQ= Health-Care Self-Determination Theory; BCT= Behaviour change technique; F&V= Fruits and vegetables; M= months; FACT-B= functional assessment of cancer therapy breast; LTEQ= Leisure-Time Exercise Questionnaire; LLFDI= late life function and disability index; PAMA= Physical Activity Maintenance Assessment; NACRT= neoadjuvant chemoradiotherapy; PASES= Physical activity Self-Efficacy Scale; PSS= Perceived Stress Scale; ACSM= American College of Sports Medicine; NRCT= Non-randomized controlled trial ; NA= Not applicable; EMA= Ecological momentary assessment; TSRQ-PQ= Treatment self-regulaion questionnaire-Physical activity; LTPAQ= Lifetime Total Physical Activity Questionnaire; BRFSS= Behavioural Risk Factor Surveillance Survey; EXSE= Exercise Self-Efficacy Scale; MOEES= Multidimensional Outcome Expectation for Exercise Scale; FSI= Fatigue Symptom Inventory; SSE= Social Support for Exercise Scale; RSES= Rosenberg Self-Esteem Scale; BSES= Barriers Self-Efficacy Scale; DBQ= Exercise Decision Balance Questionnaire; YPAS= Yale Physical Activity Survey; FACT-G= Functional Assessment of Cancer Therapy: General; AAQ= Active Australia Questionnaire; BREQ-2= Behavioural Regulation in Exercise Questionnaire-2; DBSPA= decisional balance scale for physical activity; PHQ= Patient Health Questionnaire; EBQOA= Exercise Barrier Questionnaire for Older Adults; EQoLQ= Exercise & Quality of Life Questionnaire; IPAQ= International Physical Activity Questionnaire; RAPA= Rapid Assessment of Physical Activity Scale; ASLQ= The Abbreviated Self-Leadership Questionnaire; BPAT= Brief Physical Assessment Tool; ESAS= Edmonton Symptom Assessment System; WHI-PHQ= Women’s Health Initiative Physical Activity Questionnaire; FACT-C= Functional Assessment of Cancer; Therapy – Colorectal; PAS= Perceived autonomy support; PNSE= Psychological Needs Satisfaction in Exercise; BSES= barriers self-efficacy scale; PBIS= perceived barriers interference scale; OES= outcome expectations scale; ACS= American Cancer Society; SSEHS= social support for exercise habits scale; PROMIS 2016= Patient Reported Outcomes Measurement Information System; AET= Aerobic Exercise Training; RET= Resistance exercise training; ;PSDQ‐S= Physical Self-Description Questionnaire Short Form; ESE= Exercise Self‐Efficacy scale; LTEQ= Leisure Time Exercise Questionnaire; HPAL= High physical activity level; MPAL= Moderate physical activity level; LPAL= Light physical activity level; CSs= Cancer survivors PA
