# Supplementary material for: Psychosocial correlates of physical activity in cancer survivors: a systematic review and meta-analysis
Source: J Cancer Surviv. 2024 Mar 6;19(4):1385–402. doi: 10.1007/s11764-024-01559-6 (PMC12283835; doi:10.1007/s11764-024-01559-6)
Supplement: Supplementary file 3 — Supplementary file3 (DOCX 21 KB) [file 11764_2024_1559_MOESM3_ESM.docx]

**Additional File 3 – Breast cancer results**

Breast cancer specific results

| **Predictor** | **Nº studies** | **Nº times tested** | **PA outcomes (total)** | | | **PA outcomes <6m** | | | **PA outcomes >6m** | | |
| --- | --- | --- | --- | --- | --- | --- | --- | --- | --- | --- | --- |
|  |  |  | **Positive** | **No association** | **Negative** | **Positive** | **No association** | **Negative** | **Positive** | **No association** | **Negative** |
| Exercise self-efficacy | 7 | 13 | 10 | 3 |  | 4 |  |  | 6 | 3 |  |
| Perceived barriers for exercise | 4 | 9 |  | 6 | 3 |  | 3 | 3 |  | 3 |  |
| Fatigue | 4 | 7 | 2 | 3 | 2 |  | 2 | 1 | 2 | 1 | 1 |
| Outcome expectations | 4 | 5 | 3 | 3 |  | 3 | 2 |  |  | 1 |  |
| Perceived social support (general) | 4 | 6 | 3 | 3 |  | 3 | 2 |  |  | 1 |  |
| Pros and Cons | 3 | 3 | 1 | 2 |  | 1 | 1 |  |  | 1 |  |
| Barriers self-efficacy | 3 | 3 | 3 |  |  | 3 |  |  |  |  |  |
| Quality of life | 3 | 5 | 4 | 1 |  | 2 | 1 |  | 2 |  |  |
| PA enjoyment | 2 | 7 | 6 | 1 |  | 3 | 1 |  | 3 |  |  |
| Friend support | 2 | 3 | 3 |  |  | 1 |  |  | 2 |  |  |
| Perceived behavioral control | 2 | 3 | 3 |  |  | 2 |  |  | 1 |  |  |
| Physical functioning | 2 | 3 | 3 |  |  | 3 |  |  |  |  |  |
| Bodily pain | 2 | 3 |  | 1 | 2 |  | 1 | 2 |  |  |  |
| Depression | 2 | 3 |  | 1 | 2 |  | 1 | 2 |  |  |  |
| Family participation | 1 | 6 | 5 | 1 |  | 3 |  |  | 2 | 1 |  |
| Family reward/punishment | 1 | 6 | 1 | 5 |  |  | 3 |  | 1 | 2 |  |
| Friend participation | 1 | 6 | 4 | 2 |  | 2 | 1 |  | 2 | 1 |  |
| Exercise stage of change | 1 | 6 | 6 |  |  | 3 |  |  | 3 |  |  |
| General health perceptions | 1 | 2 | 2 |  |  | 2 |  |  |  |  |  |
| Symptom burden | 1 | 2 |  | 1 | 1 |  | 1 | 1 |  |  |  |
| Intention | 1 | 1 | 1 |  |  |  |  |  | 1 |  |  |
| Instrumental attitudes | 1 | 1 | 1 |  |  |  |  |  | 1 |  |  |
| Affective attitudes | 1 | 1 |  | 1 |  |  |  |  |  | 1 |  |
| Injunctive norms | 1 | 1 |  | 1 |  |  |  |  |  | 1 |  |
| Descriptive norms | 1 | 1 |  | 1 |  |  |  |  |  | 1 |  |
| Fear/concerns related to exercise | 1 | 1 |  | 1 |  |  | 1 |  |  |  |  |
| Body image | 1 | 1 |  | 1 |  |  | 1 |  |  |  |  |
| Task self-efficacy | 1 | 1 | 1 |  |  | 1 |  |  |  |  |  |
| Family support | 1 | 1 | 1 |  |  | 1 |  |  |  |  |  |
| Advanced lower extremity function | 1 | 1 | 1 |  |  | 1 |  |  |  |  |  |
| Outcome expectancies | 1 | 1 | 1 |  |  | 1 |  |  |  |  |  |
| Behavioral capability | 1 | 1 | 1 |  |  | 1 |  |  |  |  |  |

PA= physical activity; 6m= 6 months
