# Supplementary material for: Psychosocial correlates of physical activity in cancer survivors: a systematic review and meta-analysis
Source: J Cancer Surviv. 2024 Mar 6;19(4):1385–402. doi: 10.1007/s11764-024-01559-6 (PMC12283835; doi:10.1007/s11764-024-01559-6)
Supplement: Supplementary file 4 — Supplementary file4 (DOCX 23 KB) [file 11764_2024_1559_MOESM4_ESM.docx]

**Additional file 5 – Colorectal cancer results**

Table colorectal cancer specific results

| **Predictor** | **Nº studies** | **Nº times tested** | **PA outcomes (total)** | | | **PA outcomes <6m** | | | **PA outcomes >6m** | | |
| --- | --- | --- | --- | --- | --- | --- | --- | --- | --- | --- | --- |
|  |  |  | **Positive** | **No association** | **Negative** | **Positive** | **No association** | **Negative** | **Positive** | **No association** | **Negative** |
| Fatigue | 5 | 6 | 3 | 1 | 2 | 3 | 1 | 2 |  |  |  |
| Motivation (quantity) | 2 | 3 | 2 | 1 |  | 1 | 1 |  |  | 1 |  |
| Mental health | 2 | 3 |  | 2 | 1 |  | 2 | 1 |  |  |  |
| Quality of life | 2 | 2 | 2 |  |  | 2 |  |  |  |  |  |
| Perceived social support (general) | 2 | 2 |  | 2 |  |  | 2 |  |  |  |  |
| Physical function | 2 | 2 | 2 |  |  | 2 |  |  |  |  |  |
| Rewards | 1 | 2 | 2 |  |  | 1 |  |  | 1 |  |  |
| Behavior awareness and volition | 1 | 2 | 2 |  |  | 1 |  |  | 1 |  |  |
| Constructive cognition | 1 | 2 | 2 |  |  | 1 |  |  | 1 |  |  |
| Relatedness | 1 | 2 |  | 2 |  |  | 1 |  |  | 1 |  |
| Autonomous motivation | 1 | 2 | 1 | 1 |  |  | 1 |  | 1 |  |  |
| Exercise self-efficacy | 1 | 1 | 1 |  |  | 1 |  |  |  |  |  |
| Stress | 1 | 1 |  | 1 |  |  | 1 |  |  |  |  |
| Perceived barriers for exercise | 1 | 1 |  | 1 |  |  | 1 |  |  |  |  |
| Attitude | 1 | 1 |  | 1 |  |  | 1 |  |  |  |  |
| Perceived behavioral control | 1 | 1 | 1 |  |  | 1 |  |  |  |  |  |
| Subjective norms | 1 | 1 |  | 1 |  |  | 1 |  |  |  |  |
| Physical health | 1 | 1 |  | 1 |  |  | 1 |  |  |  |  |
| Benefit of PA | 1 | 1 |  | 1 |  |  | 1 |  |  |  |  |
| PA enjoyment | 1 | 1 |  | 1 |  |  | 1 |  |  |  |  |
| Difficulty | 1 | 1 |  | 1 |  |  | 1 |  |  |  |  |
| Physical well-being | 1 | 1 | 1 |  |  | 1 |  |  |  |  |  |
| Functional well-being | 1 | 1 | 1 |  |  | 1 |  |  |  |  |  |
| Emotional well-being | 1 | 1 |  | 1 |  |  | 1 |  |  |  |  |
| Social well-being | 1 | 1 |  | 1 |  |  | 1 |  |  |  |  |
| Amotivation | 1 | 1 |  |  | 1 |  |  | 1 |  |  |  |
| Introjected regulation | 1 | 1 | 1 |  |  | 1 |  |  |  |  |  |
| Identified regulation | 1 | 1 | 1 |  |  | 1 |  |  |  |  |  |
| Intrinsic regulation | 1 | 1 | 1 |  |  | 1 |  |  |  |  |  |
| External regulation | 1 | 1 |  | 1 |  |  | 1 |  |  |  |  |
| Competence | 1 | 1 | 1 |  |  | 1 |  |  |  |  |  |
| Perceived autonomy support | 1 | 1 | 1 |  |  | 1 |  |  |  |  |  |
| Autonomy | 1 | 1 | 1 |  |  | 1 |  |  |  |  |  |
| Knowledge | 1 | 1 |  | 1 |  |  | 1 |  |  |  |  |
| Exercise stage of change | 1 | 1 | 1 |  |  | 1 |  |  |  |  |  |
| Personality | 1 | 1 |  | 1 |  |  | 1 |  |  |  |  |
| Vitality | 1 | 1 | 1 |  |  | 1 |  |  |  |  |  |
| General health perceptions | 1 | 1 | 1 |  |  | 1 |  |  |  |  |  |
| Physical role disability | 1 | 1 | 1 |  |  | 1 |  |  |  |  |  |
| Emotional role functioning | 1 | 1 |  | 1 |  |  | 1 |  |  |  |  |
| Social role functioning | 1 | 1 |  | 1 |  |  | 1 |  |  |  |  |
| Bodily pain | 1 | 1 |  | 1 |  |  | 1 |  |  |  |  |

PA= physical activity; 6m= 6 months
