# Supplementary figures and images for: Psychosocial correlates of physical activity in cancer survivors: a systematic review and meta-analysis
Source: J Cancer Surviv. 2024 Mar 6;19(4):1385–402. doi: 10.1007/s11764-024-01559-6 (PMC12283835; doi:10.1007/s11764-024-01559-6)

Additional file 5. Self-efficacy funnel plot


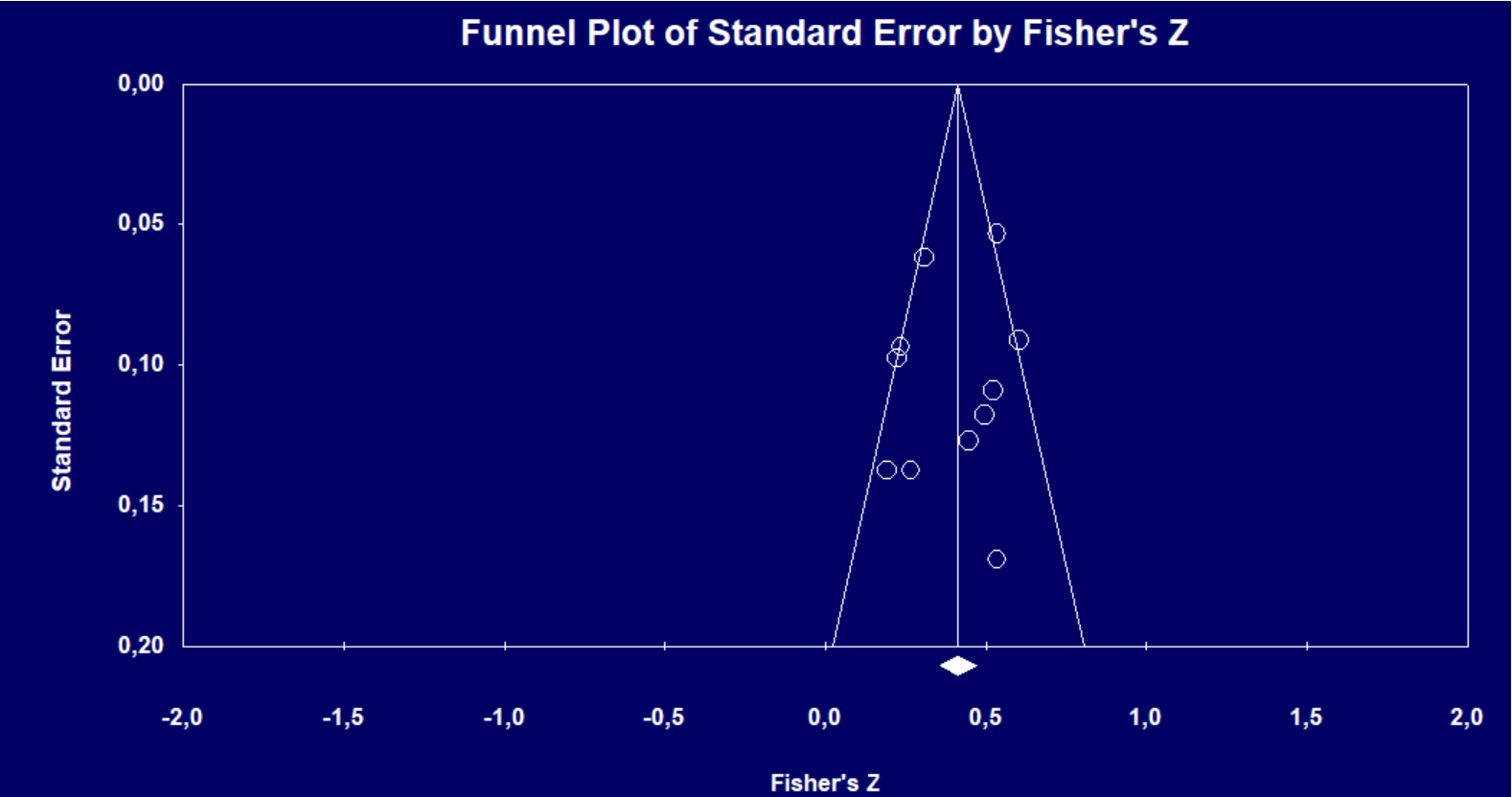

Supplement: Supplementary file 5 — Supplementary file5 (DOCX 106 KB) [file 11764_2024_1559_MOESM5_ESM.docx]
