# Supplementary material for: Psychosocial correlates of physical activity in cancer survivors: a systematic review and meta-analysis
Source: J Cancer Surviv. 2024 Mar 6;19(4):1385–402. doi: 10.1007/s11764-024-01559-6 (PMC12283835; doi:10.1007/s11764-024-01559-6)
Supplement: Supplementary file 6 — Supplementary file6 (DOCX 35 KB) [file 11764_2024_1559_MOESM6_ESM.docx]

**Additional File 6 - Quality assessment results**

Table of quality assessment results

|  | **1** | **5** | **6** | **8** | **11** | **13** | **14** | **15** | **16** | **19** | **Design** | **Blinding** | **Selection bias** | **Drop-outs** | **Confounders** | **Data collection** | **Data analysis** | **Report** | **Global rating** |
| --- | --- | --- | --- | --- | --- | --- | --- | --- | --- | --- | --- | --- | --- | --- | --- | --- | --- | --- | --- |
| Bélanger et al., 2012 | 2.i. | NA | NA | 3 | 4 | 2 | 1 | 1 | 1 | 1 | moderate | NA | weak | no rating | moderate | strong | strong | strong | moderate |
| Coups et al., 2009 | 2.i. | NA | NA | 4 | 4 | 1 | 1 | 1 | 1 | 1 | moderate | NA | weak | no rating | strong | strong | strong | strong | moderate |
| Frikkel et al., 2020 | 2.i. | NA | NA | 3 | 4 | 2 | 1 | 1 | 1 | 1 | moderate | NA | weak | no rating | moderate | strong | strong | strong | moderate |
| Hiensch et al., 2020 | 1.i. | 1 | 3 | 3 | 1 | 3 | 1 | 2 | 1 | 1 | Strong | moderate | weak | strong | weak | strong | moderate | strong | weak |
| Karvinen et al., 2008 | 2.ii. | NA | NA | 3 | 2 | 3 | 1 | 2 | 1 | 2 | moderate | NA | weak | moderate | weak | strong | moderate | moderate | weak |
| Kwarteng et al., 2020 | 1.i. | 3 | 3 | 3 | 2 | 1 | 1 | 1 | 1 | 1 | Strong | weak | weak | moderate | strong | strong | strong | strong | weak |
| Lee et al., 2018 | 2.i. | NA | NA | 3 | 4 | 2 | 1 | 1 | 1 | 1 | moderate | NA | weak | no rating | moderate | strong | strong | strong | moderate |
| Lee et al., 2020 | 2.i. | NA | NA | 3 | 4 | 3 | 1 | 1 | 1 | 1 | moderate | NA | weak | no rating | strong | strong | strong | strong | moderate |
| Lesser et al., 2021 | 2.i. | NA | NA | 3 | 4 | 4 | 1 | 2 | 1 | 1 | moderate | NA | weak | no rating | weak | strong | moderate | strong | moderate |
| Mayer et al., 2018 | 1.i. | 1 | 3 | 3 | 2 | 3 | 1 | 2 | 1 | 1 | Strong | moderate | weak | moderate | weak | strong | moderate | strong | weak |
| Mazzoni et al., 2021 | 1.ii | 1 | 3 | 3 | 2 | 2 | 1 | 1 | 1 | 1 | Strong | moderate | weak | moderate | weak | strong | strong | strong | moderate |
| Morielli et al., 2017 | 1.iii. | 2 | 2 | 3 | 1 | 3 | 1 | 2 | 1 | 1 | Strong | weak | weak | strong | weak | strong | moderate | strong | weak |
| Murray et al., 2019 | 2.i. | NA | NA | 3 | 4 | 3 | 1 | 2 | 1 | 2 | moderate | NA | weak | no rating | weak | strong | moderate | moderate | weak |
| Park et al., 2020 | 2.i. | NA | NA | 3 | 4 | 1 | 1 | 1 | 1 | 1 | moderate | NA | weak | no rating | strong | strong | strong | strong | moderate |
| Paxton et al., 2019 | 2.i. | NA | NA | 3 | 4 | 3 | 1 | 2 | 1 | 1 | moderate | NA | weak | no rating | weak | strong | moderate | strong | weak |
| Peddle et al., 2008a | 2.i. | NA | NA | 3 | 4 | 3 | 1 | 2 | 1 | 1 | moderate | NA | weak | no rating | weak | strong | moderate | strong | weak |
| Price et al., 2021 | 2.i. | NA | NA | 3 | 4 | 1 | 1 | 1 | 1 | 1 | moderate | NA | weak | no rating | strong | strong | strong | strong | moderate |
| Ribeiro et al., 2018 | 2.i. | NA | NA | 3 | 4 | 2 | 1 | 1 | 1 | 1 | moderate | NA | weak | no rating | moderate | strong | strong | strong | moderate |
| Robertson et al., 2018 | 2.i. | NA | NA | 1 | 4 | 1 | 1 | 1 | 1 | 2 | moderate | NA | strong | no rating | strong | strong | strong | moderate | strong |
| Rogers et al., 2008 | 2.i. | NA | NA | 3 | 4 | 3 | 1 | 2 | 1 | 2 | moderate | NA | weak | no rating | weak | strong | moderate | moderate | weak |
| Stevinson et al., 2009 | 2.i. | NA | NA | 3 | 4 | 3 | 1 | 2 | 1 | 2 | moderate | NA | weak | no rating | weak | strong | moderate | moderate | weak |
| Tabaczynski et al., 2019 | 2.i. | NA | NA | 3 | 4 | 2 | 1 | 1 | 1 | 1 | moderate | NA | weak | no rating | moderate | strong | strong | strong | moderate |
| Wilson et al., 2006 | 2.iii. | NA | NA | 3 | 4 | 3 | 1 | 1 | 1 | 1 | moderate | NA | weak | no rating | strong | strong | strong | strong | moderate |
| Collins et al., 2018 | 2.i. | NA | NA | 3 | 4 | 3 | 1 | 2 | 1 | 2 | moderate | NA | weak | no rating | weak | strong | moderate | moderate | weak |
| Wurz and Brunet, 2019 | 2.i. | NA | NA | 3 | 4 | 2 | 1 | 1 | 1 | 1 | moderate | NA | weak | no rating | moderate | strong | strong | strong | moderate |
| Stone et al., 2019 | 2.ii. | NA | NA | 3 | 4 | 1 | 1 | 1 | 1 | 1 | moderate | NA | weak | no rating | strong | strong | strong | strong | moderate |
| Frensham et al., 2018 | 1.i. | 2 | 1 | 3 | 1 | 2 | 1 | 1 | 1 | 1 | Strong | moderate | weak | strong | moderate | strong | strong | strong | moderate |
| Farrokhzadi et al. 2016 | 2.i. | NA | NA | 3 | 4 | 1 | 1 | 1 | 1 | 1 | moderate | NA | weak | no rating | strong | strong | strong | strong | moderate |
| Kampshoff et al., 2016 | 2.i. | NA | NA | 3 | 4 | 3 | 1 | 2 | 1 | 2 | moderate | NA | weak | no rating | weak | strong | moderate | moderate | weak |
| Lee et al., 2016 | 2.ii. | NA | NA | 3 | 1 | 3 | 1 | 2 | 1 | 1 | moderate | NA | weak | strong | weak | strong | moderate | strong | weak |
| Mama et al., 2015 | 1.i. | 3 | 3 | 3 | 3 | 2 | 1 | 1 | 1 | 1 | Strong | weak | weak | weak | moderate | strong | strong | strong | weak |
| Rogers et al., 2015 | 2.i. | NA | NA | 3 | 4 | 3 | 1 | 2 | 1 | 1 | moderate | NA | weak | no rating | weak | strong | moderate | strong | weak |
| Kang et al., 2014 | 2.i. | NA | NA | 3 | 4 | 2 | 2 | 1 | 1 | 1 | moderate | NA | weak | no rating | moderate | moderate | strong | strong | moderate |
| Short et al., 2014 | 2.i. | NA | NA | 3 | 4 | 3 | 1 | 2 | 1 | 2 | moderate | NA | weak | no rating | weak | strong | moderate | moderate | weak |
| Basen-Engquist et al., 2013 | 1.iii. | 2 | 2 | 3 | 2 | 3 | 2 | 2 | 1 | 1 | Strong | weak | weak | moderate | weak | moderate | moderate | strong | weak |
| Mosher et al., 2013 | 1.i. | 3 | 3 | 3 | 3 | 2 | 1 | 1 | 1 | 2 | Strong | weak | weak | weak | moderate | strong | strong | moderate | weak |
| Phillips and McAuley., 2013 | 2.i. | NA | NA | 3 | 4 | 3 | 1 | 2 | 1 | 2 | moderate | NA | weak | no rating | weak | strong | moderate | moderate | weak |
| Courneya et al., 2011 | 1.i. | 1 | 2 | 3 | 1 | 3 | 1 | 2 | 1 | 1 | Strong | moderate | weak | strong | weak | strong | moderate | strong | weak |
| Rogers et al., 2011 | 1.i. | 1 | 3 | 3 | 1 | 3 | 1 | 2 | 1 | 1 | Strong | moderate | weak | strong | weak | strong | moderate | strong | weak |
| Keogh et al., 2010 | 2.i. | NA | NA | 3 | 4 | 3 | 1 | 2 | 1 | 1 | moderate | NA | weak | no rating | weak | strong | moderate | strong | weak |
| Kucukvardar et al., 2021 | 2.i | NA | NA | 3 | 4 | 3 | 3 | 2 | 1 | 1 | moderate | NA | weak | no rating | weak | weak | weak | strong | weak |
| Vallance et al., 2010 | 1.i. | 1 | 3 | 3 | 1 | 3 | 1 | 2 | 1 | 1 | Strong | moderate | weak | strong | weak | strong | moderate | strong | weak |
| Finnegan et al., 2007 | 2.i. | NA | NA | 3 | 4 | 3 | 1 | 2 | 1 | 1 | moderate | NA | weak | no rating | weak | strong | moderate | strong | weak |
| Peddle et al., 2008b | 2.i. | NA | NA | 4 | 4 | 3 | 1 | 2 | 1 | 2 | moderate | NA | weak | no rating | weak | strong | moderate | moderate | weak |
| James et al., 2006 | 2.i. | NA | NA | 3 | 4 | 2 | 1 | 1 | 1 | 1 | moderate | NA | weak | no rating | moderate | strong | strong | strong | moderate |
| Culos-reed et al., 2005 | 2.ii. | NA | NA | 3 | 4 | 3 | 1 | 2 | 1 | 1 | moderate | NA | weak | no rating | weak | strong | moderate | strong | weak |
| Bennett et al., 2007 | 1.i. | 1 | 2 | 3 | 1 | 3 | 1 | 2 | 1 | 2 | Strong | moderate | weak | strong | weak | strong | moderate | moderate | weak |
| Ott et al., 2004 | 2.i. | NA | NA | 3 | 4 | 3 | 1 | 2 | 1 | 2 | moderate | NA | weak | no rating | weak | strong | moderate | moderate | weak |
| Blanchard et al., 2002 | 2.i. | NA | NA | 4 | 4 | 2 | 1 | 1 | 1 | 2 | moderate | NA | weak | no rating | moderate | strong | strong | moderate | moderate |
| Ungar et al., 2016 | 1.i. | 3 | 3 | 3 | 1 | 3 | 1 | 2 | 1 | 2 | Strong | weak | weak | strong | weak | strong | moderate | moderate | weak |
| André et al., 2018 (study 1) | 2.i. | NA | NA | 3 | 4 | 3 | 2 | 2 | 1 | 2 | moderate | NA | weak | no rating | weak | moderate | moderate | moderate | weak |
| André et al., 2018 (study 2) | 1.i. | 1 | 1 | 3 | 1 | 3 | 2 | 2 | 1 | 1 | Strong | strong | weak | strong | weak | moderate | moderate | strong | weak |
| Winger et al. 2014 | 1.i. | 1 | 3 | 3 | 4 | 2 | 1 | 1 | 1 | 1 | Strong | moderate | weak | strong | moderate | strong | strong | strong | moderate |
| Skiba et al., 2022 | 2.i | NA | NA | 1 | 4 | 1 | 4 | 1 | 1 | 2 | moderate | NA | strong | no rating | strong | no rating | strong | moderate | strong |
| Smith et al., 2018 | 2.i. | NA | NA | 4 | 4 | 1 | 2 | 1 | 1 | 1 | moderate | NA | weak | no rating | strong | moderate | strong | strong | moderate |
| Smith-Turchyn et al., 2021 | 2.i. | NA | NA | 3 | 4 | 2 | 2 | 1 | 1 | 1 | moderate | NA | weak | no rating | moderate | moderate | strong | strong | moderate |
| Karvinen et al., 2007 | 2.i. | NA | NA | 4 | 4 | 3 | 1 | 2 | 1 | 2 | moderate | NA | weak | no rating | weak | strong | moderate | moderate | weak |
| Courneya et al., 2004 | 1.i. | 1 | 1 | 3 | 1 | 3 | 1 | 2 | 1 | 1 | Strong | strong | weak | strong | weak | strong | moderate | strong | weak |
| Yan et al., 2021 | 2.i. | NA | NA | 3 | 4 | 1 | 1 | 1 | 1 | 1 | moderate | NA | weak | no rating | strong | strong | strong | strong | moderate |
| Krok-Schoen et al., 2021 | 2.i. | NA | NA | 3 | 4 | 3 | 2 | 2 | 1 | 1 | moderate | NA | weak | no rating | weak | moderate | moderate | strong | weak |
| Papadopoulos1 et al., 2022 | 1.i. | 1 | 1 | 3 | 1 | 3 | 2 | 2 | 1 | 2 | strong | strong | weak | strong | weak | moderate | moderate | moderate | weak |
| Ng at al., 2021 | 2.1. | NA | NA | 3 | 4 | 3 | 2 | 2 | 1 | 1 | moderate | NA | weak | no rating | weak | moderate | moderate | strong | weak |
| Krok-Schoen et al., 2022 | 2.i. | NA | NA | 5 | 4 | 2 | 4 | 1 | 1 | 1 | moderate | NA | no rating | no rating | moderate | no rating | strong | strong | moderate |
| Pinto et al., 2023 | 1.i. | 1 | 1 | 3 | 3 | 2 | 1 | 1 | 1 | 2 | strong | strong | weak | weak | moderate | strong | strong | moderate | weak |

NA= not applicable
