# Supplementary material for: Psychosocial correlates of physical activity in cancer survivors: a systematic review and meta-analysis
Source: J Cancer Surviv. 2024 Mar 6;19(4):1385–402. doi: 10.1007/s11764-024-01559-6 (PMC12283835; doi:10.1007/s11764-024-01559-6)
Supplement: Supplementary file 7 — Supplementary file7 (DOCX 16 KB) [file 11764_2024_1559_MOESM7_ESM.docx]

**SURE Checklist**

A - Identification, selection and appraisal of studies

A1) Were selection criteria reported?

Yes

A2) Was the search comprehensive?

Partially

A3) Is the review up-to-date?

Yes

A4) Was biased selection of articles avoided?

Yes

A5) Were appropriate criteria used to assess the risk of bias?

Yes

A6) Overall identification, selection and appraisal of studies

Reliable

B - Analysis of the findings

B1) Were characteristics and results of included studies reliably reported?

Yes

B2) Were methods used to analyse the findings reported?

Yes

B3) Was the extent of heterogeneity described?

Yes

B4) Were the findings combined (or not combined) appropriately?

Yes

B5) Were factors that could explain heterogeneity explored?

Not applicable

B6) Overall analysis of findings

Not applicable

C - Overall assessment of the reliability of the review

C1) Other considerations

No other quality issues identified

C2) Overall reliability of the review

Reliable: This is a good quality systematic review with only minor limitations
